# Supplementary material for: Clinical and cost-effectiveness of teen online problem-solving for adolescents who have survived an acquired brain injury in the UK: protocol for a randomised, controlled feasibility study (TOPS-UK)
Source: BMJ Open. 2019 Aug 22;9(8):e029349. doi: 10.1136/bmjopen-2019-029349 (PMC6707668; doi:10.1136/bmjopen-2019-029349)
Supplement: Supplementary file 1 [file bmjopen-2019-029349supp001.pdf]

ONLINE SUPPLEMENTARY APPENDIX

Qualitative interview topic guide

General Introduction

- You have been invited to take part/are taking part in the TOPS-UK feasibility study.
- If this study is successful, we would like to conduct a bigger study, but before we do that we need your views and suggestions on the best way to run it.
- It is particularly important for us to learn about your experiences while you were taking part.
- Teenagers and parents may have different experiences. We want to know how it was for both of you, and we value all your answers.
- There are no right or wrong answers.
- Participation is voluntary so if at any point you prefer not to carry on please feel free to stop the interview. We can also have a break during the interview if you wish.
- Whatever you say in this interview will be confidential and used only for this research project. I will record this interview and have it transcribed (typed out) later to keep an accurate record of your views. If anything is recorded that could reveal your identity it will not be included in the written transcription which will be anonymised. If we use any quotes from your contributions in research conferences, publications or events, nothing that could identify you will be included.
- Is it OK with you to record this interview?

**Topics for all qualitative interviews:**	**Study objective number(s)**	***Prompts***
Their experience of online consent forms	ii	*Did you feel you had enough information to make your decision to give consent? Was the consent form straightforward?*
Their experience of being randomised to treatment	x	*How did you feel about being allocated to one group?*
Their experience of online research questionnaires (some families might not have completed all of the study questionnaires)	v	*Ease of completion, clarity of questionnaires, burden of completion, and convenience of online format (any alternative suggestions). Did reminders and periodic emails help/ hinder- any suggestions to change?*

**If completed at least 1 session of the TOPS-UK intervention: Topics for teenager and parent in the intervention (TOPS-UK + TAU)**	**Study objective number(s)**	***Prompts***
Their experience of the TOPS-UK intervention programme	iv, v, xi	*How did you feel about being in this group?*<br>*How did you find the computer sessions?*<br>*How did you find the Skype sessions?*<br>*How did you get on with the coach?*
The effects of the programme on the teenager's ability to stay positive, solve problems, be organised, control their emotions		

and look after themselves.		
The effects of the programme on the parent's ability to support their teenager.		
What could be changed to improve the TOPS-UK programme?	iv	*Were there any aspects of the programme which you would change?*<br>*What aspects did you find most/least helpful?*

**Topics for teenager and parent randomised to treatment as usual (TAU)**	**Study objective number(s)**	***Prompts***
Their experience of TAU	v, x, xiv	*How did you feel about being in the control group? Did it impact on your view of the study?*
The effects of TAU on the teenager's ability to stay positive, solve problems, be organised, control their emotions and look after themselves.		
The effects of TAU on the parent's ability to support their teenager.		
What support would be helpful to families of teenagers with acquired brain injury?		*Face-to-face, telephone, online, home visits, clinic visits, which therapists/therapy would be helpful?*

**For all participants:**<br>**Topics not already covered:**	**Study Objective number(s)**	***Prompts***
What worked well and what less well in the study?	v, x,	
Is there anything that we can do to make the study more acceptable to families like yours in the future?		
Is there anything else you would like to talk about that we haven't mentioned?		
Give contact details for further information/discussion		

Ending questions
Is there anything you would like to talk more about? Have we missed anything?
Thank participants for taking time to join the telephone interview. Explain that if anyone has any follow up questions or comments they are welcome to call/ email the researcher.
