# Supplementary material for: Clinical and cost-effectiveness of teen online problem-solving for adolescents who have survived an acquired brain injury in the UK: protocol for a randomised, controlled feasibility study (TOPS-UK)
Source: BMJ Open. 2019 Aug 22;9(8):e029349. doi: 10.1136/bmjopen-2019-029349 (PMC6707668; doi:10.1136/bmjopen-2019-029349)
Supplement: Author's manuscript [file bmjopen-2019-029349.draft_revisions.pdf]

BMJ Open is committed to open peer review. As part of this commitment we make the peer review history of every article we publish publicly available.

When an article is published we post the peer reviewers' comments and the authors' responses online. We also post the versions of the paper that were used during peer review. These are the versions that the peer review comments apply to.

The versions of the paper that follow are the versions that were submitted during the peer review process. They are not the versions of record or the final published versions. They should not be cited or distributed as the published version of this manuscript.

BMJ Open is an open access journal and the full, final, typeset and author-corrected version of record of the manuscript is available on our site with no access controls, subscription charges or pay-per-view fees (http://bmjopen.bmj.com).

If you have any questions on BMJ Open's open peer review process please email info.bmjopen@bmj.com

BMJ Open

The clinical and cost effectiveness of Teen Online Problem Solving for adolescents who have survived an acquired brain injury in the UK: Protocol for a randomised, controlled feasibility study (TOPS-UK)

Journal:	*BMJ Open*
Manuscript ID	bmjopen-2019-029349
Article Type:	Protocol
Date Submitted by the Author:	22-Jan-2019

Complete List of Authors:	Limond, Jenny ; University of Exeter,<br>Wade, Shari L.; Cincinnati Childrens Hosp Med Ctr<br>Vickery, Patricia; Plymouth University, Peninsula Clinical Trials Unit<br>Jeffery, A; University of Plymouth, Peninsula Clinical Trials Unit<br>Warren, Fiona; University of Exeter Medical School, Institute of Health Research<br>Hawton, Annie; University of Exeter<br>Smithson, Janet; University of Exeter<br>Ford, Tamsin; University of Exeter<br>Haworth, Sarah; Patient Representative<br>Adlam, Anna-Lynne; University of Exeter, Psychology

Keywords:	Child and adolescent psychology, Executive function difficulty, Problem solving, Online, Randomised controlled trial

SCHOLARONE™ Manuscripts

The clinical and cost effectiveness of Teen Online Problem Solving for adolescents who have survived an acquired brain injury in the UK: Protocol for a randomised, controlled feasibility study (TOPS-UK)

Abbreviated title: Feasibility study of online problem solving tool for adolescents after a brain injury

Corresponding author:
Dr Jenny Limond
Senior Lecturer
Department of Psychology
Washington Singer Building
University of Exeter
EX4 4QQ
Email: j.limond@exeter.ac.uk
Telephone: 01392 722209

Authors:
Jenny A Limond, Psychology Department, University of Exeter, Exeter, UK
Shari L Wade, Physical Medicine and Rehabilitation, Cincinnati Children's Hospital, Cincinnati, USA
Jane Vickery, Peninsula Clinical Trials Unit, Plymouth University, Plymouth, UK
Alison N Jeffery, Peninsula Clinical Trials Unit, Plymouth University, Plymouth, UK
Fiona C Warren, University of Exeter Medical School, Exeter, UK
Annie Hawton, University of Medical School, Exeter, Exeter, UK
Janet Smithson, Psychology Department, University of Exeter, Exeter, UK
Tamsin Ford, University of Exeter Medical School, Exeter, UK
Sarah Haworth, Patient and Participant Representative, Exeter, UK
Anna-Lynne R Adlam, Psychology Department, University of Exeter, Exeter, UK

Word count (excluding title page, abstract, references, figures and tables)
6327

For peer review only

For peer review only - http://bmjopen.bmj.com/site/about/guidelines.xhtml

Abstract

Introduction
Paediatric acquired brain injury is a leading cause of mortality in children in the UK. Improved treatment during the acute phase has led to increased survival rates, albeit with life-long morbidity in terms of social and emotional functioning. This is the protocol for a feasibility randomised controlled trial with an embedded qualitative study and feasibility economic evaluation. If feasible, a later definitive trial will test the effectiveness and cost-effectiveness of an online intervention to enhance problem solving ability versus treatment as usual.

Methods and analysis
Fifty adolescents and their families identified by primary or secondary care clinicians at participating UK National Health Service Trusts will be recruited and individually randomised in a 1:1 ratio to receive the online intervention or treatment as usual. Participants will be followed up by online questionnaires 17 weeks after randomisation to capture acceptability of the study and intervention and resource use data. Qualitative interviews will capture participants' and clinicians' experiences of the study.

Ethics and dissemination
This study has been granted ethical approval by the South West-Exeter Research Ethics Committee (ref 17/SW/0083). Results will be disseminated via peer-reviewed publications and will inform the design of a larger trial.

Trial registration number ISRCTN10906069

Key words
Child and adolescent psychology, executive function, problem solving, online, randomised controlled trial

Strengths and limitations of this study

- This is the first study to test an online problem solving tool for adolescents who have survived a brain injury in the UK.
- The study explores the feasibility of recruitment strategies, data collection and economic evaluation to inform the design of a larger randomised controlled trial.
- The effect of the online intervention on executive functioning is not assessed.
- This study is conducted online, with minimal telephone support and no face-to-face contact.

INTRODUCTION

In 2012, paediatric acquired brain injury (pABI) was identified as one of the leading causes of death in children aged 5-19 years[1]. In the UK, 280 children per 100,000 require at least 24 hours hospitalization for traumatic brain injury (TBI) each year[2]. When considering other aetiologies such as brain tumours, stroke, and infection, there are even greater numbers of children surviving pABI[3].The long-term, even life-long, effects on social functioning, cognition, emotions and behaviour mean that pABI is a leading cause of disability.[4,5]

Despite these on-going difficulties, children with pABI do not automatically receive specialist education, often returning to mainstream schools with little or no additional support. Furthermore, although specialist tertiary NHS services do exist in the UK, there are limited outreach support or community services for ongoing and emerging difficulties[6]. Families report struggling to access

For peer review only - http://bmjopen.bmj.com/site/about/guidelines.xhtml

appropriate treatments despite their child developing significant and complex needs. Families also report significant distress and burden when caring for a child who has survived pABI, leading to an increased risk of mental health difficulties in parents and siblings, and a breakdown in parental relationships[7]. Without appropriate neuropsychological interventions, pABI can lead to increased risk of substance misuse[8], mental health difficulties[9], unemployment and criminal behaviour in adulthood[10]. Thus, the long-term costs of pABI to the individual, his/her family and society as a whole can be substantial[11].

Executive function (EF) difficulties (higher-order cognitive processes that govern goal-directed action and adaptive responses to novel or complex situations) are common following pABI[12]. These difficulties can present later in childhood (particularly in early adolescence), sometimes many years after the initial brain injury. EF difficulties can have far reaching effects, including problems with academic achievement[13], social communication[14], emotion and behaviour regulation[15], and peer relationships[16]. Indeed, families caring for children with pABI often report that EF difficulties significantly contribute to their increased levels of stress[17]. Effective interventions targeting child EF and family burden are likely to have significant patient benefit especially as developmental studies show that poor family function can also negatively impact on the child's development of EF abilities[18].

Despite the clear negative impact of EF difficulties following pABI, there is currently a paucity of research examining the effectiveness of interventions to improve function[19]. In an attempt to address this gap, Wade and colleagues developed an online web-based problem solving, communication and self-regulation intervention for adolescents (12–17 years) who have survived a TBI, and their families (Teen Online Problem Solving, TOPS)[20]. Research to date has been undertaken in the United States, and has focused on children and adolescents who have survived a TBI and their families[20-26]. These studies have demonstrated improvements in EF, child behaviour, parental depression and family-child conflict when comparing TOPS with an internet-resource comparison control. Families have reported finding the online delivery of the intervention helpful, making the intervention easy to access at a time that is convenient for them. This is an important consideration when providing interventions for children with pABI in the UK and their families, because brain injury services in the UK often cover a large geographical region (many accepting national referrals), and families often have multiple commitments when caring for these teenagers with complex needs.

Despite this emerging evidence base, it is not yet known whether the gains described can also be demonstrated in adolescents with non-traumatic brain injuries (e.g. brain tumour, stroke), when the intervention is delivered remotely (i.e. no initial home visit to set up the intervention thus keeping delivery costs to a minimum), and if the intervention is cost-effective relative to treatment as usual (TAU).

A British version of the TOPS programme, (TOPS-UK) has been developed. The didactic material is presented with British English spelling and narration, UK-based information including scenarios, video clips and resource links. TOPS-UK has been further modified to include examples of those with paediatric acquired brain injury as well as traumatic brain injury.

The potential of TOPS-UK to have a positive effect on the lives of thousands of adolescents with pABI needs testing in a rigorous multi-centre, randomised, controlled trial (RCT) in the UK, allowing a definitive test of its clinical and cost effectiveness. This feasibility study is designed to determine if a large RCT is feasible, with the ultimate aim of the research being to provide an evidence base for effective neuropsychological interventions to be recommended by the Department of Health guidelines (e.g. NICE) for survivors of pABI.

A two-arm randomised controlled feasibility phase II study will be conducted[27]. The aim is to assess

For peer review only - http://bmjopen.bmj.com/site/about/guidelines.xhtml

whether a larger, fully powered, definitive randomised controlled trial (RCT) and cost-effectiveness analysis can be successfully planned and delivered. In order to gain as much information as possible to inform a fully powered RCT, additional objectives are considered in this feasibility trial, including accessing information related to treatment set-up, delivery costs and resource use (Table 1).

Table 1 Study objectives

Number	Study objectives
1	Clinician ability to identify participants
2	Feasibility of online screening, consent and randomisation process
3	Number of participants at each site identified at each of the following time points: eligible, approached, consented, completed screening, completed baseline questionnaires, randomised, completed treatment, completed outcome assessments
4	Adolescent and parent acceptability of the intervention and outcome measures
5	Parents' and adolescents' experiences of study participation
6	Means and standard deviations for full RCT potential primary outcome measure
7	Estimate of the resources required to run main trial
8	Identification of any sites that might not be suitable for inclusion in the fully powered trial (eg due to poor recruitment)
9	Evaluation of appropriateness of the trial and economic evaluation methods and procedures
10	Assessment of participants' willingness to be randomised and complete outcome measures
11	Assessment of participants' adherence to intervention (number of sessions completed)
12	Assessment of participants' completion of outcome measures
13	Assessment of loss to follow-up
14	Coach's experience of supporting intervention delivery
15	Adolescent and parent experiences of TAU (TAU arm)

METHODS AND ANALYSIS

This is a randomised, controlled, multi-centre feasibility study in young patients with pABI aged 12-18 years. Fifty participants will be randomised (minimised by site and type of brain injury) in a 1:1 ratio to receive TAU or treatment as usual plus Teen Online Problem Solving (TAU+TOPS-UK). Treatment allocation will not be blinded. The protocol (version 4.1, 30 August 2018) is registered on Current Controlled Trials ISRCTN10906069 and follows Standard Protocol Items, Recommendations for Interventional Trial (SPIRIT) guidelines[28].

Setting

Two settings will be utilised to examine feasibility outcomes associated with recruitment of adolescents known to have had a pABI:

1. Direct referral by clinicians of adolescents who are under the outpatient care (usually neurology, neuropsychology or paediatric) of five participating NHS Trusts in England. Screening logs will be maintained at all sites to record the number of patients screened and enrolled in the study.

For peer review only - http://bmjopen.bmj.com/site/about/guidelines.xhtml

2. Recruitment from Participant Identification Centres (PIC) in primary or secondary care, or charitable organisations. Potential participants will respond directly to study promotion including leaflets/posters/participant information sheets distributed by parent-led groups, charities or support groups.

Study participation will be supported by research nurses at each participating NHS Trust. For participants directly referred to the study, nurses will make the first approach to the family. For those identified through PICs, families will be asked to contact the research nurse whose details are provided on the patient information sheet (PIS). The research nurse will support all families during the online consent and will be a point of contact for all participants during the study. The research nurse or other research team member will support baseline and follow-up measure completion. The intervention will be provided through online materials and video conference links with a coach trained in the TOPS-UK intervention. The coach will also receive weekly supervision from a qualified clinical psychologist on the research team to ensure treatment fidelity. The single study coach will be centrally based at the lead site.

Participant inclusion criteria

To be included in the study, participants will be aged 12-18 years at the time of recruitment and have survived a pABI. Diagnosis of pABI includes: moderate to severe TBI (Glasgow Coma Scale score <12 and/or post-traumatic amnesia > 24 hours and/or loss of consciousness >30 minutes), stroke, brain tumour, central nervous system infection (fungal, bacterial protozoal or viral origin including encephalitis, meningitis, brain abscess, tuberous sclerosis, acute disseminated encephalomyelitis and Guillain-Barre syndrome). Participants must be medically stable (having reached a plateau in recovery following the index injury or illness). They must also have access to the internet, and have executive function difficulties in the opinion of the local Principal Investigator (PI). At least one parent or guardian living with the adolescent must be available for the family to participate. Adolescents with a co-morbid diagnosis of attention deficit hyperactivity disorder, autistic spectrum disorder or specific learning difficulties (e.g. reading difficulties including dyslexia) will be eligible to be recruited.

Participant exclusion criteria

Participants will be excluded from the study for the following reasons: insufficient English language, capacity or willingness for the parent/adolescent to consent/assent to the study; pre-injury or co-morbid conditions such as sensory impairments and global developmental delay, known to impair engagement with the computer and treatment materials; or non-accidental brain injury.

Participant recruitment

The trial complies with the Declaration of Helsinki and ICH Good Clinical Practice (GCP) guidelines. At sites, the local PI, research nurse or other member of the research team in conjunction with the clinical team will identify potential eligible participants, usually from current outpatient clinic lists or review of medical records. An anonymised log of all participants screened with reasons for exclusion will be kept at site. Information sheets for both adolescents and their parents/guardians will be provided to potentially eligible families. The relevant PIS will be given in person to families attending an outpatient clinic, or sent by post with a cover letter from the recruiting clinician. All families will be provided with contact details and informed that a member of the research team will contact them by telephone at least 24 hours after receipt of the information to discuss the study.

During this telephone call, the parent and adolescent will be given the opportunity to ask questions about the study and to confirm whether they are interested in participating. If interested, eligibility criteria will be checked with the parent to confirm suitability for the study and ascertain which parent will participate in the intervention and complete parent-rated outcomes. A summary of what to expect from the on-line screening and consent process will also be provided.

For peer review only - http://bmjopen.bmj.com/site/about/guidelines.xhtml

For peer review only

The process of explaining the study and determining eligibility of potential participants will be undertaken by an appropriately trained member of the research team as delegated by the PI, depending on local arrangements. All staff undertaking this process for this study must have current Good Clinical Practice training and must be authorised by the PI to explain the study and assess eligibility, on the site's study delegation log.

Online Screening and Consent

Informed consent and assent will be obtained via the study-specific website, developed and maintained by the UK Clinical Research Collaboration (UKCRC)-registered Peninsula Clinical Trials Unit (CTU) at Plymouth University.

Consent/assent

The consent process will depend upon the age of the participants:

- Participants aged 16-18 years will provide online informed consent
- Participants aged 12-15 years will provide online, informed assent. Those who have given assent and who reach the age of 16 years during the study, will be asked to provide online informed consent
- Parents of all participants aged 12-15 years will provide online informed consent on behalf of their child ('signature' from one parent required)
- All participating parents will provide online informed consent for their own participation in the study

Web-based consent process

The research nurse will enter on the study website brief details of those families who wish to participate in the study and who satisfy the initial inclusion/exclusion criteria. This will trigger an email to the adolescent, containing a link to the age-appropriate assent or consent form within the website, which the adolescent should complete.

Following completion of the adolescent assent/consent form, a separate email will be sent to the nominated parent participant containing a link to the parental consent form. Parents of adolescents aged 12-15 years will be asked to consent on behalf of their adolescent in addition to giving consent for their own participation. Two reminder emails will be sent from CTU followed by a 'last chance' email, all with reminders of the study link. If the family has not completed the consent process after two weeks, the local research team will be notified and will contact the family to ask if they still wish to participate in the study.

Face-to-face consent option

If the parent and/or adolescent has not completed the online consent process, CTU will inform the local research nurse who will telephone the family to offer the option of a clinic visit when the nurse can support the parent and/or adolescent to complete online consent/assent.

Online BRIEF-2 parent-rating and allocation of study number

Following completion of the consent process, the parent will be prompted to complete the online Behaviour Rating Inventory of Executive Function (BRIEF-2[29]) parent-rating questionnaire. If the parent does not wish to complete this immediately following the consent process, it will be possible to return to the website to complete it later. Two reminder emails will be sent from CTU followed by a 'last chance' email as above. Face-to face support to complete the BRIEF-2 will also be offered by the research nurse, as above. The parent must complete all elements of the BRIEF-2 before progressing to the baseline measures as this is the proposed primary outcome measure for a definitive trial.

For peer review only - http://bmjopen.bmj.com/site/about/guidelines.xhtml

For peer review only

The website will calculate the BRIEF-2 scores. The website will assign a unique 4-digit study number to each participant and participants will be identified in all study-related documentation by this study number. An email to both the adolescent and the parent will then be sent, inviting them to follow a web link to complete the baseline measures, and informing them of their study number.

As part of the consent process, adolescents and parents will be reminded via the website that they are free to withdraw from the study at any time without giving a reason and without affecting further treatment.

Baseline data collection (adolescent and parent self-completion)
On receiving the link to the baseline measures page of the study website, the adolescent and parent will complete their separate baseline questionnaires. The baseline measures should be completed within two weeks; if either the parent or adolescent have not undertaken these after one week, an email reminder to the parent (in all cases) and the adolescent (if adolescent measures not done) will be sent. In addition to the questionnaires, the parent will provide sociodemographic information, details of the adolescent's past medical history and concurrent medication.

Participants will also be given the option of completing baseline measures with telephone support from a member of the research team. A £15 gift voucher will be sent to each family upon completion of their baseline measures.

Randomisation
Participants will be randomised via a web-based system created by the CTU in conjunction with the trial statistician, using minimisation by study site and type of brain injury (TBI/tumour/other). Participants will be randomly allocated in a 1:1 ratio to receive either treatment as usual (TAU) or TOPS-UK + TAU. The minimisation process will retain a stochastic element to retain allocation concealment. If any participant is found to be ineligible following randomisation, he/she will be excluded from analyses.

This study is not blinded. Following randomisation, the adolescent, parent, PI and research nurse will be notified by email of the adolescent's treatment allocation, and this information will be held on the study site file. The parent and adolescent will also be advised when to expect further contact from the research team. In addition, the TOPS coach will be notified of all participants allocated to the intervention arm (TOPS-UK + TAU).

Baseline data collection (research nurse)
The research nurse or another member of the research team at each site will record relevant injury characteristics data for each adolescent (including premorbid/developmental history) in the study-specific web-based case report form (CRF).

Recording study participation in medical notes
The research nurse will make a record of study participation in the adolescent's hospital notes according to local practice, stating that consent was obtained online and face-to-face support was given if appropriate. The research nurse will file a copy of the PIS in the hospital notes along with printed evidence from the study website of the informed consent process and who was involved. The nurse will also send a standard letter to the participant's GP recording study participation.

INTERVENTION
The study has two treatment arms, 'TAU' and 'TAU+TOPS-UK'. Those allocated to the TAU+TOPS-UK arm will work through the intervention programme taking 1-2 weeks per module. The coach will coordinate the timing of the modules so that all families will have completed the intervention at 16 weeks. This will allow time for other commitments and holidays/breaks to be built into the schedule.

For peer review only - http://bmjopen.bmj.com/site/about/guidelines.xhtml

The length of time taken to complete each module will be recorded to inform timing of follow-up in the main trial.

Treatment as usual (control)

TAU will vary at each recruitment site because currently there is no evidence-based treatment for adolescents with pABI and their families. The type of TAU received will be recorded for each participant at baseline via a parent-rated measure of adolescent health and social care resource use (a modified version of the Client Service Receipt Inventory). There will be no opportunity for any TAU participants to receive the TOPS-UK intervention at any point during or after the study.

Teen Online Problem Solving – TOPS (intervention*)*

TOPS is an online intervention, which is provided by the Cincinnati Children's Hospital website, via a link from the study website. For the purposes of this study, the US-based TOPS treatment content was edited to include British (English) spelling, narration and UK resource links, and modified to suit participants with pABI rather than traumatic brain injury only. All participants allocated to the TAU+TOPS-UK arm will be supported by a single TOPS-UK coach who will make weekly contact by video conference.

For those participants allocated to TAU+TOPS-UK, the TOPS-UK coach will contact the parent and/or adolescent by telephone, introduce themselves, discuss how to access the treatment modules on the website and how to log in for subsequent video call sessions. The coach will email these participants a 'start-up' pack describing how to access the online materials.

Ten subsequent sessions (Table 2) consisting of self-guided didactic content regarding problem solving skills, video clips modelling these skills, and exercises to practise the skills, will then be completed every 7-10 days by the family and the adolescent with pABI. The online modules should take 30 minutes to complete, with the video call sessions taking 60 minutes. The parent will work together with the adolescent to complete the sessions. When the family has reviewed the materials for each session, the TOPS-UK coach will conduct a video call with the adolescent and parent who agreed to participate in the study. During this session they will review the online materials and practise the problem solving skills using a problem identified by the family/adolescent. They will then plan the next session and agree a suitable time for the video call. Ideally, video calls will be held weekly, but this period can be increased as agreed through discussions between the coach and the family.

For all families allocated to TAU+TOPS-UK, the TOPS-UK coach will record details in the study database relating to intervention compliance e.g. number and date of Skype sessions completed, length of each session, progress/engagement of adolescent and parent.

Table 2 TOPS-UK sessions (complete 10 in total)

**Core sessions (complete all five)**	Getting started and staying positive
	Steps of problem solving
	Getting organised
	Staying in control
	Taking care of you
**Tailored sessions (choose four)**	Dealing with fatigue
	Managing fear and worry
	Controlling your anger and improving communication
	Listening, talking and reading non-verbal cues
	Social behaviour and joining a group
	Working with the school
**Core final session**	Bringing it all together

For peer review only - http://bmjopen.bmj.com/site/about/guidelines.xhtml

Completion of all ten modules is expected to take each family 16 weeks in total. If the programme has not been completed after 16 weeks the intervention will be discontinued at that point. Study follow-up will proceed as if the programme had been completed.

Sample Size Calculation
The study will aim to screen 20 potential participants at each site (n=100 in total), and aim to recruit a sample size of 50 participants from five sites. This should provide sufficient data to assess the feasibility and acceptability of the study. Although the sample size for the full RCT will be estimated based on the minimum clinically important difference (MCID) for the BRIEF score[30] (5-points), the standard deviation for this patient population is currently unknown. A sample size of 50 is considered a realistic target and would be sufficient to address the feasibility aims (i.e. acceptability of questionnaires etc), as well as aiming to provide at least 12 participants reporting quantitative baseline data to calculate the required standard deviations[31].

Data Management
The CTU data management team is responsible for data management. Each participant will be allocated a unique trial number on consenting to participate and will be identified in all study-related documentation by the trial number and initials. A record of names and addresses linked to participants' trial numbers will be maintained by the research nurses at each site for administrative purposes and stored securely. This is an online study, with no requirement for data entry at CTU. Functions within the study website will remind participants to complete online measures, and flag up missing fields. However, participants will be able to progress through a questionnaire leaving data fields unfilled. The SQL Server database will be designed and maintained by the CTU data programming teams. Access by researchers will be password protected. In order to avoid problems with mislaid usernames and passwords, participants will access the web pages through links emailed to them by CTU. Once a web page has been completed by the participant it will be locked to prevent further data entry.

Confidentiality
All data will be collected and managed in accordance with the Data Protection Act 1998. Each participant will be allocated a unique study number and will be identified in all study-related documentation by their study number and initials. All data will be entered on a password-protected SQL Server database and encrypted using a stored procedure. After all data cleaning has been performed and the database locked, anonymised data will be exported to the trial statistician.

Data Analysis Plan
All randomised participants will be included in the analyses according to their randomised allocation, irrespective of adherence to treatment in the TOPS-UK arm or receipt of treatment in the TAU arm. No imputation of missing baseline or follow-up data will be performed. The study is not sufficiently powered to detect a significant treatment effect with regard to clinical or cost-effectiveness and thus a formal comparison will not be undertaken. The reported analyses will therefore be restricted to descriptive statistics on the outcome measures with appropriate point estimates (mean, median, percentage, etc.), standard deviations, and 95% confidence intervals for between group differences. For the questionnaire outcomes, approaches to missing individual items will be in accordance with the guidelines for missing item procedures for each questionnaire. Where no guidelines for individual missing items are available, the mean of the completed items will be used to replace missing items if 10% or fewer are missing. Statistical analyses will be performed following final data cleaning and locking of the dataset. No interim analyses are planned. All analyses will be performed using Stata v.14 and will be performed by a statistician using a dataset with treatment allocation masked.

Assessment of trial feasibility
The primary feasibility outcomes of this study include: (i) number of participants at each stage of the

For peer review only - http://bmjopen.bmj.com/site/about/guidelines.xhtml

study, at each site, with stages including: identified as potentially eligible, approached, consented to study, completed screening and baseline, randomised, completed treatment, and completed outcome assessments; (ii) identification of any sites that might not be suitable for inclusion in the fully powered trial (e.g. due to poor recruitment); (iii) evaluation of appropriateness of the trial and economic evaluation methods and procedures; (iv) assessment of participants' willingness to be randomised to treatment allocation; (v) adherence to treatment assessed as number of sessions completed, a session will be recorded as complete if the participant has been through every page, reached the end and completed the coach web-linked session related to that material; and (vi) attrition (failure to complete outcome measures at follow-up).

The further outcome is evaluation of the standard deviation of the BRIEF-2 (parent) score for this patient population. This is the proposed primary outcome for the main trial and required for calculation of the sample size.

Measurement of outcomes

This feasibility study aims to evaluate all aspects of the proposed fully powered RCT and cost-effectiveness analysis including recruitment and retention numbers, and completion of outcome measures proposed for the main trial. Outcomes will be assessed by questionnaires and interviews. Table 3 provides a summary of outcome measures for this feasibility trial.

Table 3. Summary of Outcome Measures

Outcome group	Outcome measure	Objective	Evaluation time point(s)
Primary outcome (main trial)	Parent BRIEF-2	6	Baseline, 17 weeks post-randomisation
Secondary outcomes (main trial)	Adolescent: RCADS, SDQ, CBQ, BRIEF-2, CHU9D<br>Adult: RCADS, SDQ, EQ5D-5L, PHQ9, GAD7, CBQ, CSRI	1,10,11	Baseline, 17 weeks post-randomisation
Measures of adherence (intervention group)	Participation in weekly Skype sessions.<br>Record of login to intervention website (frequency, duration, progression)	3,11	Data recorded by coach.<br>Data captured automatically by database throughout 16 week intervention period.
Intervention feasibility and acceptability (intervention group)	Qualitative interviews with families<br>Intervention acceptability questionnaires (adolescent and parent)<br>Qualitative interview with coach	1-4, 6, 9	End of trial<br>17 weeks post-randomisation<br>End of trial
Experience of TAU (TAU arm)	Qualitative interviews with families	15	End of trial
Study acceptability (both groups)	Study participation questionnaires (adolescent and parent)<br>Qualitative interviews (adolescent and parent)	5	End of trial

Abbreviations:

BRIEF-2 Behaviour Rating Inventory of Executive Function, 2nd Edition

RCADS Revised Child and Anxiety Depression Scale

For peer review only - http://bmjopen.bmj.com/site/about/guidelines.xhtml

SDQ	Strengths and Difficulties Questionnaire
CBQ	Conflict Behaviour Questionnaire
CHU9D	Child Health Utility 9D
EQ5D-5L	EuroQol 5 Dimensions 5 Levels Questionnaire
PHQ9	Patient Health Questionnaire 9
GAD7	Generalised Anxiety Disorder 7-item
CSRI	Client Service Receipt Inventory

Outcome measures

These are detailed in Table 4. The proposed primary outcome for the main trial is the BRIEF-2[29], a parent and self-report measure of everyday executive function skills. Secondary outcomes will include parent reports of their child's health and behavior, using the Revised Child Anxiety and Depression Scale[32] (RCADS) and Strengths and Difficulties Questionnaire[33] (SDQ) to evaluate the adolescents' social, emotional and behavioural functioning.

A mixed methods approach (questionnaires and interviews) will be used to address outcomes relating to adolescent and parent acceptability of the intervention and outcome measures, coach's experience of supporting intervention delivery, parents' and adolescents' experiences of study participation, assessment of participants' willingness to be randomised to treatment and complete outcome measures, evaluation of appropriateness of the trial and economic evaluation methods and procedures, and adolescent and parent experiences of TAU (TAU arm).

Parental reports of their own and family quality of life, health, emotional functioning and family interactions will be evaluated using the EuroQol 5 dimensions questionnaire[34] (EQ-5D-5L), the Patient Health Questionnaire[35] (PHQ-9), the Generalised Anxiety Disorder[36] (GAD-7) questionnaire and the Conflict Behaviour Questionnaire[37] (CBQ).

Use of health and social care resources will be assessed through parental report on the Modified Client Service Receipt Inventory[38] (CSRI). Adolescents involved in the study will also complete questionnaires relating to their executive function skills (BRIEF-2[29] adolescent version), quality of life (Child health-related quality of life[39]; CHU-9D), emotional functioning (RCADS[32]), social, emotional and behaviour functioning (SDQ[33]) and their family interactions (CBQ[37]).

The EQ-5D-5L and CHU-9D also provide data on quality-adjusted life-years (QALYs) which can be used in cost-effectiveness analyses.

Table 4 Trial Schedule

Study procedure	Screening	Baseline	16 week intervention period	Follow-up 17 weeks
Consent/assent (adolescent +/- parents)	X			
Consent (parents)	X			
Characteristics of ABI (CRF)	X			
Current medication (adolescent)		X		X
BRIEF parent-rating	X			X
**Parents**				

For peer review only

For peer review only - http://bmjopen.bmj.com/site/about/guidelines.xhtml

Demographics, past medical history		X		
SDQ parent-rated		X		X
PHQ-9		X		X
GAD-7		X		X
CBQ		X		X
EQ5D-5L		X		X
CSRI		X		X
RCADS parent version		X		X
**Adolescents**				
Consent/assent (participants +/- parents)	X			
BRIEF-2		X		X
RCADS		X		X
SDQ		X		X
CHU-9D		X		X
CBQ		X		X
**Both parent and adolescent**				
Treatment adherence				X
Treatment acceptability rating				X
Study participation feedback				X
Qualitative telephone interviews (all)				X

Follow-up questionnaires

Adolescents and their parents in both treatment arms will be sent an email asking them to complete their online follow-up outcome measures at 17 weeks post-randomisation. In the event of non-completion, at least two reminders will be sent by email, followed by a reminder telephone call from the site research team if required. As with the baseline measures, participants will be given the option (at consent/assent) to complete the online follow-up measures with telephone support from a member of the research team. If participants do not complete the follow-up measures within four weeks they will receive a 'last chance' email with a reminder of the study link. A £15 gift voucher will be sent to each family following completion of the follow-up questionnaires.

QUALITATIVE INTERVIEWS

A single qualitative researcher will conduct semi-structured qualitative process evaluation interviews by telephone or video call with all participants (including those who have not completed follow-up questionnaires but who have not formally withdrawn from the study), research nurses and the study coach. PIS relating to the qualitative interviews will be emailed in advance and consent will be sought at the beginning of the interview by the qualitative researcher. Adolescent and parent participants will be offered the option of being interviewed together or separately. Interviews will be conducted with the help of an agreed topic guide (see online supplementary appendix) and are expected to last between 20-60 minutes. Interviews will be audio-recorded and transcribed verbatim. The transcribed interview data will be fully anonymised and any demographic data about participants will be stored separately. Qualitative interview data will be managed using a computer software package such as Nvivo 11 and thematically analysed[40]. The analysis and results will be checked/validated by a second qualitative researcher. The following interviews will be conducted:

review only

For peer review only - http://bmjopen.bmj.com/site/about/guidelines.xhtml

1. **All participants** (both groups). These interviews will allow participants to describe their whole experience of participating in the study including their willingness to be randomised and their experiences and acceptability of outcome measures, what worked well and what less well. This will cover aspects such as clarity of PIS, acceptability and ease of completing the online consent process, ease of completing online questionnaires and the parental support required the adolescent to participate and remain engaged. The interviews will explore adolescent and parent experiences of TAU or TAU+TOPS-UK, including parent's perceptions of the impact of TAU/TAU+TOPS-UK on their ability to support the adolescent, adolescent's and parent's perceptions of the impact of TAU/TAU+TOPS-UK on adolescent's ability to stay positive, solve problems, be organised, control their emotions and look after themselves (self-care), and participants' views on potential improvements to TAU/TAU+TOPS-UK. In addition, participants in the TAU+TOPS-UK will be asked about their experience of working with the coach via video calls. This information will be helpful when planning the full RCT and implementation studies. After completion of the interview, each family will be sent a £15 gift voucher as a token of appreciation.

2. **Interview with TOPS-UK coach.** A single interview will explore the coach's experiences of supporting the TOPS-UK intervention. The interview will be held by telephone, or face to face, once every participant allocated to the intervention arm has completed the programme.

3. **Interviews with research nurses.** All research nurses involved with the study at the five participating sites will be invited to participate in a single interview to explore their experiences of supporting families through the consent process and any support required to complete baseline or follow-up measures. Feedback on methods of contact with families, number of contact attempts made, how much support was required from adolescents and/or parents, barriers to recruitment and suggestions for future studies will also be sought.

Participants who withdraw from the study, participants who register their interest but do not complete the study consent forms, and participants who discontinue the intervention and who do not complete the follow-up questionnaires (i.e., non-adherent but not explicitly withdrawn), will be invited to complete an anonymous online feedback survey. The survey will invite participants to comment on any aspects of the study that they found difficult, any aspects of the study that they liked, and suggestions on how the study can be improved.

ECONOMIC EVAUATION

This feasibility study will be used to develop a framework for a subsequent, policy-relevant, cost-effectiveness analysis to be undertaken alongside a future RCT. Economic evaluation methods will be developed and assessed regarding the collection of resource use, cost, and outcome data. Data on resource use associated with the set-up and delivery of the TOPS treatment will be collected at the participant and coach level e.g. coach contact and non-contact time per participant, equipment and consumable costs, training and supervision requirements for the TOPS-UK coach. Data regarding service use will be collected from all participants' parents using a version of the CSRI specifically modified for this population. This includes use of mental health, community rehabilitation, and neuropsychology and educational psychology services and has been previously trailed in this population (Wilson et al., in preparation). The study will also consider the most appropriate manner in which to capture data on health-related quality of life for use in the estimation of the cost per QALY (quality-adjusted life year) of the TOPS-UK treatment. The feasibility of participants self-completing the CHU-9D and parents completing the EQ-5D-5L, will be assessed.

In addition to this primary economic outcome, the appropriateness of considering the cost-per unit change on other relevant indicators of health status collected in the baseline and follow-up phases

For peer review only - http://bmjopen.bmj.com/site/about/guidelines.xhtml

will be explored.

SAFETY REPORTING

The risks associated with participating in this study are considered minimal. There is a slight chance for those in the intervention group that raising awareness of injury-related cognitive or behavioural problems through communication and problem solving might increase family burden and possibly contribute to conflict between family members. However, the purpose of the intervention is ultimately to equip families with skills to handle these difficulties by learning how to change the way they solve problems and talk with one another, and the TOPS-UK coach supporting the online intervention will be trained to handle any emerging problems. Should any issues arise, the coach will have access to a qualified clinical psychologist to provide further support and advice. There is therefore no requirement to report non-serious adverse events in this study, although serious adverse events (SAEs) will be monitored.

SAEs may be reported by clinicians or researchers at site, the TOPS-UK coach, by participants themselves or by any other informant. Adverse event data will be monitored by the Trial Steering Committee (TSC) to ensure safety. The TSC includes an independent statistician, psychologist, paediatrician and patient and public representative and will meet on approximately four occasions. All suspected SAEs will be reported within 24 hours of discovery to the CTU who will notify the Chief Investigator. All SAEs will be followed up until resolution.

PARTICIPANT AND PUBLIC INVOLVEMENT

A PPI group including patients and the CI was convened at an early stage of grant development and trial design and continued to meet during study set up. The group gave feedback on the patient-facing materials including age-appropriate PIS, informed consent forms and qualitative interview topic guide content. A lay-representative who is Director of Services and Innovation at the national Child Brain Injury Trust (CBIT) is a co-applicant to the grant, and a member of both the Trial Management Group (TMG) and TSC. During the recruitment period, this lay representative has been liaising with local CBIT representatives and PICs to identify potentially eligible families. A study twitter account will post trial updates to promote public engagement with the study. The twitter site currently follows and is followed by national brain injury charities disseminating trial updates to a wide audience.

Another PPI representative (parent of a child with pABI) is a member of the TMG, regularly attending meetings, and freely contributing to the discussions. Both members provide advice and suggest solutions to problems encountered during the trial, with particular expertise in barriers to recruitment and communication issues within families with a teenager with pABI. This expertise has been cascaded to site staff through a research nurse forum.

The contribution of PPI members within the TMG will be valuable during analysis, interpretation and dissemination of the study results.

STUDY MANAGEMENT AND OVERSIGHT

The study sponsor organisation is the Royal Devon and Exeter NHS Foundation Trust, Barrack Road, Exeter EX2 5DW. Day to day trial management is administered through the UKCRC-registered Peninsula Clinical Trials Unit at Plymouth University. A Trial Management Group including the Chief Investigator, CTU trial managers, trial statistician and other personnel relevant to the study (e.g. clinicians, CTU data manager, patient and Sponsor representatives) will meet regularly (usually monthly) throughout the duration of the trial to oversee practical management of the trial.

For peer review only - http://bmjopen.bmj.com/site/about/guidelines.xhtml

A TSC, chaired by an independent member, will oversee the conduct and safety of the trial, ensuring that milestones are achieved and general scientific probity is maintained. A Data Monitoring Committee was not required for this feasibility study.

ETHICS AND DISSEMINATION

The study will be undertaken at acute NHS Trusts, subject to appropriate Research Ethics Committee (REC) and Health Research Authority (HRA) approvals. The trial will be conducted in accordance with the protocol, the principles of the Declaration of Helsinki and ICH GCP. Any amendments of the protocol will be submitted to the REC for approval. On request the Chief/Principal Investigator should make available relevant trial-related documents for monitoring and audit by the Sponsor or the relevant Research Ethics Committee.

The study team will prepare a plain English summary of the study results which will be sent to the study participants as soon as possible after the end of the study. The final results of the study will be disseminated via presentations at appropriate scientific meetings and conferences and publication in appropriate peer-reviewed journals. The data from this study will be used to inform the design and accompanying grant application for a fully powered RCT should the study be considered feasible.

DISCUSSION

In our opinion, TOPS-UK has genuine potential to have a profound and positive effect on the lives of thousands of adolescents with brain injury. The intervention itself already exists and has demonstrated efficacy in the United States, including gains in executive function (planning, problem-solving), reductions in behaviour and mood difficulties, and reductions in family burden and stress. TOPS-UK urgently requires testing in a rigorous multi-centre randomised trial in the UK, allowing a definitive test of its clinical and cost effectiveness.

The importance of the current study is to determine if a large RCT is feasible (e.g., recruitment, access to resource use data, appropriate outcome measures etc). In addition, although the study is not powered to detect effectiveness, individual patients participating in the study might gain direct benefit from the treatment (TAU+TOPS) in terms of increased executive function skills, reduced behaviour and mood difficulties, and improved quality of life. The feasibility study might also have some implications on clinical and public health practice by raising awareness of pABI, the need for effective treatment, and the variability of TAU (via the dissemination workshop and reports), and improve the measurements of quality of life and health status in adolescents with pABI (via conferences). If the current study demonstrates feasibility, outcomes will be used to inform the development of a fully powered phase III RCT to examine effectiveness and cost-effectiveness of TOPS-UK.

If the phase III RCT is successful, then the clinical and public health practice developments are potentially far reaching. For example, in addition to the direct benefits to the adolescent and his or her family, TOPS-UK might also prevent considerable NHS costs in the future by reducing healthcare service use. Furthermore, given the increased risks of criminal behaviour and poor educational and vocational outcomes following pABI, often associated with poor executive functioning, TOPS-UK might have wider social benefits for these individuals, and society as a whole. In terms of potential impact on local policy-making and improvement in service delivery, TOPS-UK is potentially very cheap to deliver because it is web-based and involves regular but brief professional support that is delivered remotely (via video calls). Furthermore, given its web-based delivery that can be accessed at a time that is convenient for the adolescent and their family, TOPS-UK is less disruptive than clinic appointments, which are often delivered during school-hours. This is an important consideration for an individual with pABI because, typically, the adolescents have already missed a significant amount of school and struggle academically. By enabling free use of the materials to the NHS, clinical

For peer review only - http://bmjopen.bmj.com/site/about/guidelines.xhtml

services might routinely provide TOPS-UK to adolescents with pABI and their families, meeting the needs of multiple families simultaneously. TOPS-UK might also provide a first-line of treatment for families experiencing distress and, therefore form part of a 'stepped-care' model of service delivery such that, families who continue to experience significant difficulties, or who are at high risk, can be referred to specialist services in a timely manner. The ultimate aim of the research programme is to provide an evidence-base for effective neuropsychological interventions to be recommended by the Department of Health guidelines (e.g., NICE) for survivors of pABI.

References

1. ONS. Office for National Statistics. Children, Education and Skills: Measuring National Well-being http://wwwonsgovuk/ons/taxonomy/indexhtml?nscl=Children%2C+Education+and+ Skills. 2012.
2. Hawley CA, Ward AB, Long J, Owen DW and Magnay AR. Prevalence of traumatic brain injury amongst children admitted to hospital in one health district: a population-based study. Injury. 2003;34:256-60.
3. Hayes L, Shaw S, Pearce MS, et al., Requirements for and current provision of rehabilitation services for children after severe acquired brain injury in the UK: a population-based study. Archives of Disease in Childhood. 2017;102:813-820.
4. WHO. World Report on Child Injury Prevention. 2008.
5. Fleminger S and Ponsford J. Long term outcome after traumatic brain injury. BMJ. 2005;331:1419-20.
6. All-Party Parliamentary Group on Acquired Brain Injury. Acquired brain injury and neurorehabilitation: time for change. www.ukabif.org.uk/campaigns/appg-report. 2018
7. Tomlin P, Clarke M, Robinson G and Roach J. Rehabilitation in severe head injury in children: outcome and provision of care. Dev Med Child Neurol. 2002;44:828-37.
8. McKinlay A, Corrigan J, Horwood L and Fergusson D. Substance Abuse and Criminal Activities Following Traumatic Brain Injury in Childhood, Adolescence and Early Adulthood. J Head Trauma Rehabil. 2013.
9. Max JE. Neuropsychiatry of pediatric traumatic brain injury. Psychiatr Clin North Am. 2014;37:125-40.
10. Williams WH, Mewse AJ, Tonks J, Mills S, Burgess CN and Cordan G. Traumatic brain injury in a prison population: prevalence and risk for re-offending. Brain Inj. 2010;24:1184-8.
11. Anderson V, Spencer-Smith M, Leventer R, Coleman L, Anderson P, Williams J, Greenham M and Jacobs R. Childhood brain insult: can age at insult help us predict outcome? Brain. 2009;132:45-56.
12. Anderson V, Spencer-Smith M, Coleman L, Anderson P, Williams J, Greenham M, Leventer RJ and Jacobs R. Children's executive functions: are they poorer after very early brain insult. Neuropsychologia. 2010;48:2041-50.
13. Best JR, Miller PH and Naglieri JA. Relations between Executive Function and Academic Achievement from Ages 5 to 17 in a Large, Representative National Sample. Learn Individ Differ. 2011;21:327-336.
14. Struchen MA, Clark AN, Sander AM, Mills MR, Evans G and Kurtz D. Relation of executive functioning and social communication measures to functional outcomes following traumatic brain injury. NeuroRehabilitation. 2008;23:185-98.
15. Jurado MB and Rosselli M. The elusive nature of executive functions: a review of our current understanding. Neuropsychol Rev. 2007;17:213-33.
16. Gracey F, Watson S, McHugh M, Swan A, Humphrey A and Adlam A-L. Age of injury, emotional problems and executive functioning in understanding disrupted social relationships following childhood ABI. J Social Care and Neurodisability. 2014;5:10.

For peer review only - http://bmjopen.bmj.com/site/about/guidelines.xhtml

17. Kurowski BG, Taylor HG, Yeates KO, Walz NC, Stancin T and Wade SL. Caregiver ratings of long-term executive dysfunction and attention problems after early childhood traumatic brain injury: family functioning is important. PM R. 2011;3:836-45.
18. Hughes CH and Ensor RA. How do families help or hinder the emergence of early executive function? New Dir Child Adolesc Dev. 2009;2009:35-50.
19. Ross KA, Dorris L and McMillan T. A systematic review of psychological interventions to alleviate cognitive and psychosocial problems in children with acquired brain injury. Dev Med Child Neurol. 2011;53:692-701.
20. Wade SL, Wolfe CR and Pestian JP. A web-based family problem-solving intervention for families of children with traumatic brain injury. Behav Res Methods Instrum Comput. 2004;36:261-9.
21. Wade SL, Walz NC, Carey JC and Williams KM. Preliminary efficacy of a Web-based family problem-solving treatment program for adolescents with traumatic brain injury. J Head Trauma Rehabil. 2008;23:369-77.
22. Wade SL, Oberjohn K, Burkhardt A and Greenberg I. Feasibility and preliminary efficacy of a web-based parenting skills program for young children with traumatic brain injury. J Head Trauma Rehabil. 2009;24:239-47.
23. Wade SL, Walz NC, Carey J, Williams KM, Cass J, Herren L, Mark E and Yeates KO. A randomized trial of teen online problem solving for improving executive function deficits following pediatric traumatic brain injury. J Head Trauma Rehabil. 2010;25:409-15.
24. Wade SL, Walz NC, Carey J, McMullen KM, Cass J, Mark E and Yeates KO. Effect on behavior problems of teen online problem-solving for adolescent traumatic brain injury. Pediatrics. 2011;128:e947-53.
25. Wade SL, Walz NC, Carey J, McMullen KM, Cass J, Mark E and Yeates KO. A randomized trial of teen online problem solving: efficacy in improving caregiver outcomes after brain injury. Health Psychol. 2012;31:767-76.
26. Wade S, Stancin T, Kirkwood M, Brown T, McMullen K and Taylor H. Counselor-assisted problem solving (CAPS) improves behavioural outcomes in older adolescents with complicated mild to severe TBI. J Head Trauma Rehabil. 2014;29:10.
27. Craig P, Dieppe P, Macintyre S, et al. Developing and evaluating complex interventions: the new Medical Research Council guidance. BMJ2008;337:a1655
28. Chan A-W, Tetzlaff JM, Altman DG, Laupacis A, Gøtzsche PC, Krleža-Jerić K, Hróbjartsson A, Mann H, Dickersin K, Berlin J, Doré C, Parulekar W, Summerskill W, Groves T, Schulz K, Sox H, Rockhold FW, Rennie D, Moher D. SPIRIT 2013 Statement: Defining standard protocol items for clinical trials. *Ann Intern Med* 2013;158:200-207.
29. Gioia G, Isquith P, Guy S and Kenworthy L. Behavior Rating Inventory of Executive Function Second Edition (BRIEF 2). Psychological Assessment Resources. 2015.
30. Kurowski BG, Wade SL, Kirkwood MW, Brown TM, Stancin T and Taylor HG. Online problem-solving therapy for executive dysfunction after child traumatic brain injury. Pediatrics. 2013;132:e158-66.
31. Julious S. Sample size of 12 per group rule of thumb for pilot study. Pharmaceutical Statistics. 2005;4:5.
32. Chorpita BF, Moffitt CE and Gray J. Psychometric properties of the Revised Child Anxiety and Depression Scale in a clinical sample. Behav Res Ther. 2005;43:309-22.
33. Goodman R. The extended version of the Strengths and Difficulties Questionnaire as a guide to child psychiatric caseness and consequent burden. J Child Psychol Psychiatry. 1999;40:791-9.
34. Herdman M, Gudex C, Lloyd A, Janssen M, Kind P, Parkin D, Bonsel G and Badia X. Development and preliminary testing of the new five-level version of EQ-5D (EQ-5D-5L). Qual Life Res. 2011;20:1727-36.
35. Kroenke K, Spitzer RL and Williams JB. The PHQ-9: validity of a brief depression severity measure. J Gen Intern Med. 2001;16:606-13.

For peer review only

36. Spitzer RL, Kroenke K, Williams JB and Löwe B. A brief measure for assessing generalized anxiety disorder: the GAD-7. Arch Intern Med. 2006;166:1092-7.
37. Robin A and Foster S. Negotiating parent-adolescent conflict: A behavioural family systems approach. New York: Guildford Press; 1989.
38. Beecham J and Knapp M. Costing psychiatric interventions. London: Gaskill; 1992.
39. Stevens M, Clark A, Sander A, Mills M, Evans G and Kurtz D. Assessing the performance of a new generic measure of health related quality of life for children and refining it for use in health state valuation. Applied Health Economics and Health Policy. 2011;9:12.
40. Braun V, Clarke, V. Using thematic analysis in psychology. Qualitative Research in Psychology. 2006;3:77-101.

ACKNOWLEDGEMENTS
The authors are grateful for the support of the study sponsor (Royal Devon and Exeter NHS Foundation Trust) and the South West Peninsula NIHR Clinical Research Network. We acknowledge the contribution made by Dr Richard Tomlinson, Consultant Paediatrician, Royal Devon and Exeter NHS Foundation Trust who was a grant co-applicant and for his support during study set- up. We are also indebted to Theresa Pass, former Director of the Child Brain Injury Trust who was a grant co-applicant, and to Louise Wilkinson and members of the Child Brain Injury Trust for their continued support and advice during recruitment and study delivery.

AUTHORS' CONTRIBUTIONS
All authors except AJ and SH were co-applicants on the NIHR RfPB grant application and as such were involved in the design of this feasibility study. All authors contributed to successive drafts of this paper. JL was the CI for the first year of the study and led on developing the intervention, finalising the protocol and drafting this manuscript. SW designed the study intervention and contributed to study design. JV contributed to the study design and oversaw trial management. AJ is the trial manager, responsible for the day-to-day running of the trial, and contributed to drafts of this manuscript. FW is the trial statistician and provided expertise in the overall design of the trial. AH was responsible for the design and analysis of the economic evaluation component. JS was responsible for the design and analysis of the qualitative component. TF contributed to study design and provided advice and guidance on study delivery. SH contributed to trial management from a PPI perspective. AA was the lead grant applicant and took over as CI from JL for the second year of the study, providing clinical expertise, and drafting and revising this manuscript.

DATA SHARING
After the end of the study, information collected may be made available as an anonymised participant level dataset to other researchers under an appropriate data sharing agreement.

FUNDING and DISCLAIMER
This paper presents independent research funded by the National Institute for Health Research (NIHR) under its Research for Patient Benefit (RfPB) Programme (Grant Reference Number PB-PG-0614-34081). The views expressed are those of the author(s) and not necessarily those of the NHS, the NIHR or the Department of Health and Social Care. The funder has no role in study design or the collection, management, analysis or interpretation of data.

COMPETING INTERESTS
None declared.

Provenance and peer review Not commissioned; externally peer reviewed.

For peer review only - http://bmjopen.bmj.com/site/about/guidelines.xhtml

ONLINE SUPPLEMENTARY APPENDIX

Qualitative interview topic guide

General Introduction

- You have been invited to take part/are taking part in the TOPS-UK feasibility study.
- If this study is successful, we would like to conduct a bigger study, but before we do that we need your views and suggestions on the best way to run it.
- It is particularly important for us to learn about your experiences while you were taking part.
- Teenagers and parents may have different experiences. We want to know how it was for both of you, and we value all your answers.
- There are no right or wrong answers.
-
- Participation is voluntary so if at any point you prefer not to carry on please feel free to stop the interview. We can also have a break during the interview if you wish.
- Whatever you say in this interview will be confidential and used only for this research project. I will record this interview and have it transcribed (typed out) later to keep an accurate record of your views. If anything is recorded that could reveal your identity it will not be included in the written transcription which will be anonymised. If we use any quotes from your contributions in research conferences, publications or events, nothing that could identify you will be included.
- Is it OK with you to record this interview?

**Topics for all qualitative interviews:**	**Study objective number(s)**	***Prompts***
Their experience of online consent forms	ii	*Did you feel you had enough information to make your decision to give consent? Was the consent form straightforward?*
Their experience of being randomised to treatment	x	*How did you feel about being allocated to one group?*
Their experience of online research questionnaires (some families might not have completed all of the study questionnaires)	v	*Ease of completion, clarity of questionnaires, burden of completion, and convenience of online format (any alternative suggestions). Did reminders and periodic emails help/ hinder- any suggestions to change?*

**If completed at least 1 session of the TOPS-UK intervention: Topics for teenager and parent in the intervention (TOPS-UK + TAU)**	**Study objective number(s)**	***Prompts***
Their experience of the TOPS-UK intervention programme	iv, v, xi	*How did you feel about being in this group? How did you find the computer sessions? How did you find the Skype sessions? How did you get on with the coach?*
The effects of the programme on the teenager's ability to stay		

For peer review

positive, solve problems, be organised, control their emotions and look after themselves.		
The effects of the programme on the parent's ability to support their teenager.		
What could be changed to improve the TOPS-UK programme?	iv	*Were there any aspects of the programme which you would change?*<br>*What aspects did you find most/least helpful?*

**Topics for teenager and parent randomised to treatment as usual (TAU)**	**Study objective number(s)**	***Prompts***
Their experience of TAU	v, x, xiv	*How did you feel about being in the control group? Did it impact on your view of the study?*
The effects of TAU on the teenager's ability to stay positive, solve problems, be organised, control their emotions and look after themselves.		
The effects of TAU on the parent's ability to support their teenager.		
What support would be helpful to families of teenagers with acquired brain injury?		*Face-to-face, telephone, online, home visits, clinic visits, which therapists/therapy would be helpful?*

**For all participants:**<br>**Topics not already covered:**	**Study Objective number(s)**	***Prompts***
What worked well and what less well in the study?	v, x,	
Is there anything that we can do to make the study more acceptable to families like yours in the future?		
Is there anything else you would like to talk about that we haven't mentioned?		
Give contact details for further information/discussion		

Ending questions
Is there anything you would like to talk more about? Have we missed anything?

Thank participants for taking time to join the telephone interview. Explain that if anyone has any follow up questions or comments they are welcome to call/ email the researcher.

For peer review only

Reporting checklist for protocol of a clinical trial.

Based on the SPIRIT guidelines.

Instructions to authors

Complete this checklist by entering the page numbers from your manuscript where readers will find each of the items listed below.

Your article may not currently address all the items on the checklist. Please modify your text to include the missing information. If you are certain that an item does not apply, please write "n/a" and provide a short explanation.

Upload your completed checklist as an extra file when you submit to a journal.

In your methods section, say that you used the SPIRIT reporting guidelines, and cite them as:

Chan A-W, Tetzlaff JM, Altman DG, Laupacis A, Gøtzsche PC, Krleža-Jerić K, Hróbjartsson A, Mann H, Dickersin K, Berlin J, Doré C, Parulekar W, Summerskill W, Groves T, Schulz K, Sox H, Rockhold FW, Rennie D, Moher D. SPIRIT 2013 Statement: Defining standard protocol items for clinical trials. Ann Intern Med. 2013;158(3):200-207

		Reporting Item	Page Number
Title	#1	Descriptive title identifying the study design, population, interventions, and, if applicable, trial acronym	1
Trial registration	#2a	Trial identifier and registry name. If not yet registered, name of intended registry	2

For peer review only

Trial registration: data set	#2b	All items from the World Health Organization Trial Registration Data Set	N/A
Protocol version	#3	Date and version identifier	N/A
Funding	#4	Sources and types of financial, material, and other support	18
Roles and responsibilities: contributorship	#5a	Names, affiliations, and roles of protocol contributors	1, 18
Roles and responsibilities: sponsor contact information	#5b	Name and contact information for the trial sponsor	14
Roles and responsibilities: sponsor and funder	#5c	Role of study sponsor and funders, if any, in study design; collection, management, analysis, and interpretation of data; writing of the report; and the decision to submit the report for publication, including whether they will have ultimate authority over any of these activities	14
Roles and responsibilities: committees	#5d	Composition, roles, and responsibilities of the coordinating centre, steering committee, endpoint adjudication committee, data management team, and other individuals or groups overseeing the trial, if applicable (see Item 21a for data monitoring committee)	15
Background and rationale	#6a	Description of research question and justification for undertaking the trial, including summary of relevant	2-4

For peer review only

For peer review only - http://bmjopen.bmj.com/site/about/guidelines.xhtml

		studies (published and unpublished) examining benefits and harms for each intervention	
Background and rationale: choice of comparators	#6b	Explanation for choice of comparators	7
Objectives	#7	Specific objectives or hypotheses	4
Trial design	#8	Description of trial design including type of trial (eg, parallel group, crossover, factorial, single group), allocation ratio, and framework (eg, superiority, equivalence, non-inferiority, exploratory)	4
Study setting	#9	Description of study settings (eg, community clinic, academic hospital) and list of countries where data will be collected. Reference to where list of study sites can be obtained	4-5
Eligibility criteria	#10	Inclusion and exclusion criteria for participants. If applicable, eligibility criteria for study centres and individuals who will perform the interventions (eg, surgeons, psychotherapists)	5
Interventions: description	#11a	Interventions for each group with sufficient detail to allow replication, including how and when they will be administered	7-9
Interventions: modifications	#11b	Criteria for discontinuing or modifying allocated interventions for a given trial participant (eg, drug dose	9

For peer review only

		change in response to harms, participant request, or improving / worsening disease)	
Interventions: adherance	#11c	Strategies to improve adherence to intervention protocols, and any procedures for monitoring adherence (eg, drug tablet return; laboratory tests)	12
Interventions: concomitant care	#11d	Relevant concomitant care and interventions that are permitted or prohibited during the trial	N/A
Outcomes	#12	Primary, secondary, and other outcomes, including the specific measurement variable (eg, systolic blood pressure), analysis metric (eg, change from baseline, final value, time to event), method of aggregation (eg, median, proportion), and time point for each outcome. Explanation of the clinical relevance of chosen efficacy and harm outcomes is strongly recommended	10-12
Participant timeline	#13	Time schedule of enrolment, interventions (including any run-ins and washouts), assessments, and visits for participants. A schematic diagram is highly recommended (see Figure)	11-12
Sample size	#14	Estimated number of participants needed to achieve study objectives and how it was determined, including clinical and statistical assumptions supporting any sample size calculations	9
Recruitment	#15	Strategies for achieving adequate participant enrolment to reach target sample size	N/A

For peer review only

Allocation: sequence generation	#16a	Method of generating the allocation sequence (eg, computer-generated random numbers), and list of any factors for stratification. To reduce predictability of a random sequence, details of any planned restriction (eg, blocking) should be provided in a separate document that is unavailable to those who enrol participants or assign interventions	7
Allocation concealment mechanism	#16b	Mechanism of implementing the allocation sequence (eg, central telephone; sequentially numbered, opaque, sealed envelopes), describing any steps to conceal the sequence until interventions are assigned	7
Allocation: implementation	#16c	Who will generate the allocation sequence, who will enrol participants, and who will assign participants to interventions	7
Blinding (masking)	#17a	Who will be blinded after assignment to interventions (eg, trial participants, care providers, outcome assessors, data analysts), and how	N/A
Blinding (masking): emergency unblinding	#17b	If blinded, circumstances under which unblinding is permissible, and procedure for revealing a participant's allocated intervention during the trial	N/A
Data collection plan	#18a	Plans for assessment and collection of outcome, baseline, and other trial data, including any related processes to promote data quality (eg, duplicate measurements, training of assessors) and a description of study	7,9,12

For peer review only

		instruments (eg, questionnaires, laboratory tests) along with their reliability and validity, if known. Reference to where data collection forms can be found, if not in the protocol	
Data collection plan: retention	#18b	Plans to promote participant retention and complete follow-up, including list of any outcome data to be collected for participants who discontinue or deviate from intervention protocols	12
Data management	#19	Plans for data entry, coding, security, and storage, including any related processes to promote data quality (eg, double data entry; range checks for data values). Reference to where details of data management procedures can be found, if not in the protocol	9
Statistics: outcomes	#20a	Statistical methods for analysing primary and secondary outcomes. Reference to where other details of the statistical analysis plan can be found, if not in the protocol	9
Statistics: additional analyses	#20b	Methods for any additional analyses (eg, subgroup and adjusted analyses)	N/A
Statistics: analysis population and missing data	#20c	Definition of analysis population relating to protocol non-adherence (eg, as randomised analysis), and any statistical methods to handle missing data (eg, multiple imputation)	9
Data monitoring: formal committee	#21a	Composition of data monitoring committee (DMC); summary of its role and reporting structure; statement of	15

For peer review only

		whether it is independent from the sponsor and competing interests; and reference to where further details about its charter can be found, if not in the protocol. Alternatively, an explanation of why a DMC is not needed	
Data monitoring: interim analysis	#21b	Description of any interim analyses and stopping guidelines, including who will have access to these interim results and make the final decision to terminate the trial	N/A
Harms	#22	Plans for collecting, assessing, reporting, and managing solicited and spontaneously reported adverse events and other unintended effects of trial interventions or trial conduct	14
Auditing	#23	Frequency and procedures for auditing trial conduct, if any, and whether the process will be independent from investigators and the sponsor	N/A
Research ethics approval	#24	Plans for seeking research ethics committee / institutional review board (REC / IRB) approval	2,15
Protocol amendments	#25	Plans for communicating important protocol modifications (eg, changes to eligibility criteria, outcomes, analyses) to relevant parties (eg, investigators, REC / IRBs, trial participants, trial registries, journals, regulators)	14,15
Consent or assent	#26a	Who will obtain informed consent or assent from potential trial participants or authorised surrogates, and how (see Item 32)	6

For peer review only

Consent or assent: ancillary studies	#26b	Additional consent provisions for collection and use of participant data and biological specimens in ancillary studies, if applicable	N/A
Confidentiality	#27	How personal information about potential and enrolled participants will be collected, shared, and maintained in order to protect confidentiality before, during, and after the trial	9
Declaration of interests	#28	Financial and other competing interests for principal investigators for the overall trial and each study site	18
Data access	#29	Statement of who will have access to the final trial dataset, and disclosure of contractual agreements that limit such access for investigators	18
Ancillary and post trial care	#30	Provisions, if any, for ancillary and post-trial care, and for compensation to those who suffer harm from trial participation	N/A
Dissemination policy: trial results	#31a	Plans for investigators and sponsor to communicate trial results to participants, healthcare professionals, the public, and other relevant groups (eg, via publication, reporting in results databases, or other data sharing arrangements), including any publication restrictions	15,16
Dissemination policy: authorship	#31b	Authorship eligibility guidelines and any intended use of professional writers	15

For peer review only

Dissemination policy: reproducible research	#31c	Plans, if any, for granting public access to the full protocol, participant-level dataset, and statistical code	N/A
Informed consent materials	#32	Model consent form and other related documentation given to participants and authorised surrogates	N/A
Biological specimens	#33	Plans for collection, laboratory evaluation, and storage of biological specimens for genetic or molecular analysis in the current trial and for future use in ancillary studies, if applicable	N/A

The SPIRIT checklist is distributed under the terms of the Creative Commons Attribution License CC-BY-ND 3.0. This checklist can be completed online using https://www.goodreports.org/, a tool made by the EQUATOR Network in collaboration with Penelope.ai

For peer review only

BMJ Open

The clinical and cost effectiveness of Teen Online Problem-Solving for adolescents who have survived an acquired brain injury in the UK: Protocol for a randomised, controlled feasibility study (TOPS-UK)

Journal:	*BMJ Open*
Manuscript ID	bmjopen-2019-029349.R1
Article Type:	Protocol
Date Submitted by the Author:	05-Apr-2019

Complete List of Authors:	Limond, Jenny ; University of Exeter,<br>Wade, Shari L.; Cincinnati Childrens Hosp Med Ctr<br>Vickery, Patricia; Plymouth University, Peninsula Clinical Trials Unit<br>Jeffery, A; University of Plymouth, Peninsula Clinical Trials Unit<br>Warren, Fiona; University of Exeter Medical School, Institute of Health Research<br>Hawton, Annie; University of Exeter<br>Smithson, Janet; University of Exeter<br>Ford, Tamsin; University of Exeter<br>Haworth, Sarah; Patient Representative<br>Adlam, Anna-Lynne; University of Exeter, Psychology

**Primary Subject Heading**:	Rehabilitation medicine
Secondary Subject Heading:	Paediatrics
Keywords:	Child and adolescent psychology, Executive function difficulty, Problem solving, Online, Randomised controlled trial

SCHOLARONE™ Manuscripts

The clinical and cost effectiveness of Teen Online Problem-Solving for adolescents who have survived an acquired brain injury in the UK: Protocol for a randomised, controlled feasibility study (TOPS-UK)

Abbreviated title: Feasibility study of online problem-solving tool for adolescents after a brain injury

Corresponding author:
Dr Jenny Limond
Senior Lecturer
Department of Psychology
Washington Singer Building
University of Exeter
EX4 4QQ
Email: j.limond@exeter.ac.uk
Telephone: 01392 722209

Authors:
Jenny A Limond, Psychology Department, University of Exeter, Exeter, UK
Shari L Wade, Physical Medicine and Rehabilitation, Cincinnati Children's Hospital, Cincinnati, USA
Jane Vickery, Peninsula Clinical Trials Unit, Plymouth University, Plymouth, UK
Alison N Jeffery, Peninsula Clinical Trials Unit, Plymouth University, Plymouth, UK
Fiona C Warren, University of Exeter Medical School, Exeter, UK
Annie Hawton, University of Medical School, Exeter, Exeter, UK
Janet Smithson, Psychology Department, University of Exeter, Exeter, UK
Tamsin Ford, University of Exeter Medical School, Exeter, UK
Sarah Haworth, Patient and Participant Representative, Exeter, UK
Anna-Lynne R Adlam, Psychology Department, University of Exeter, Exeter, UK

Word count (excluding title page, abstract, references, figures and tables)
7002

For peer review only

Abstract

Introduction
Paediatric acquired brain injury is a leading cause of mortality in children in the UK. Improved treatment during the acute phase has led to increased survival rates, albeit with life-long morbidity in terms of social and emotional functioning. This is the protocol for a feasibility randomised controlled trial with an embedded qualitative study and feasibility economic evaluation. If feasible, a later definitive trial will test the effectiveness and cost-effectiveness of an online intervention to enhance problem solving ability versus treatment as usual.

Methods and analysis
Twenty-five adolescents and their families identified by primary or secondary care clinicians at participating UK National Health Service Trusts will be recruited and individually randomised in a 1:1 ratio to receive the online intervention or treatment as usual. Participants will be followed up by online questionnaires 17 weeks after randomisation to capture acceptability of the study and intervention and resource use data. Qualitative interviews will capture participants' and clinicians' experiences of the study.

Ethics and dissemination
This study has been granted ethical approval by the South West-Exeter Research Ethics Committee (ref 17/SW/0083). Results will be disseminated via peer-reviewed publications and will inform the design of a larger trial.

Trial registration number ISRCTN10906069

Key words
Child and adolescent psychology, executive function, problem-solving, online, randomised controlled trial

Strengths and limitations of this study

- This is the first study to test an online problem-solving tool for adolescents who have survived a brain injury in the UK.
- The study explores the feasibility of recruitment strategies, data collection and economic evaluation to inform the design of a larger randomised controlled trial.
- The effect of the online intervention on executive functioning is not assessed.
- This study is conducted online, with minimal telephone support and no face-to-face contact.
- This feasibility study was conducted over a short time period thus limiting the opportunity for a long-term follow-up. The definitive trial would include a follow-up period of at least 6 months.

For peer review only

INTRODUCTION

In 2012, paediatric acquired brain injury (pABI) was identified as one of the leading causes of death in children aged 5-19 years[1]. In the UK, 280 children per 100,000 require at least 24 hours hospitalization for traumatic brain injury (TBI) each year[2]. When considering other aetiologies such as brain tumours, stroke, and infection, there are even greater numbers of children surviving pABI[3].The long-term, even life-long, effects on social functioning, cognition, emotions and behaviour mean that pABI is a leading cause of disability.[4,5]

Despite these on-going difficulties, children with pABI do not automatically receive specialist education, often returning to mainstream schools with little or no additional support. Furthermore, although specialist tertiary NHS services do exist in the UK, there are limited outreach support or community services for ongoing and emerging difficulties[6]. Families report struggling to access appropriate treatments despite their child developing significant and complex needs. Families also report significant distress and burden when caring for a child who has survived pABI, leading to an increased risk of mental health difficulties in parents and siblings, and a breakdown in parental relationships[7]. Without appropriate neuropsychological interventions, pABI can lead to increased risk of substance misuse[8], mental health difficulties[9], unemployment and criminal behaviour in adulthood[10]. Thus, the long-term costs of pABI to the individual, his/her family and society as a whole can be substantial[11].

Executive function (EF) difficulties (higher-order cognitive processes that govern goal-directed action and adaptive responses to novel or complex situations) are common following pABI[12]. These difficulties can present later in childhood (particularly in early adolescence), sometimes many years after the initial brain injury. EF difficulties can have far reaching effects, including problems with academic achievement[13], social communication[14], emotion and behaviour regulation[15], and peer relationships[16]. Indeed, families caring for children with pABI often report that EF difficulties significantly contribute to their increased levels of stress[17]. Effective interventions targeting child EF and family burden are likely to have significant patient benefit especially as developmental studies show that poor family function can also negatively impact on the child's development of EF abilities[18].

Despite the clear negative impact of EF difficulties following pABI, there is currently a paucity of research examining the effectiveness of interventions to improve function[19]. In an attempt to address this gap, Wade and colleagues developed an online web-based problem-solving, communication and self-regulation intervention for adolescents (12–17 years) who have survived a TBI, and their families (Teen Online Problem-Solving, TOPS)[20]. The development of online family problem-solving treatment for pediatric TBI was predicated on evidence of the reciprocity between child recovery and parental psychological well-being and informed by input from survivors of TBI and their families. Problem-solving therapy provides a systematic approach for coping more effectively with life's challenges by creating a positive mind set and developing, implementing, and evaluating solutions to problems[21]. Online family problem-solving treatment simplifies the ABCDEs of problem-solving: defining the Aim, Brainstorming, Choosing the best strategy to implement, Doing it (creating a concrete, step by step plan), and Evaluating whether it worked. Given the focus on the family rather than the individual, TOPS also focuses on communication skills and collaborative problem-solving. For adolescents with executive function and problem-solving deficits following TBI, the steps of problem-solving also provide an executive function heuristic[22]. Additionally, the program teaches stress management and metacognitive strategies such as stopping to think before acting and self-monitoring that can serve to promote self-regulation. Similar to the face-to-face, multifamily Brain Injury Family Intervention[23], online modules provide didactic information regarding the cognitive and behavioral consequences of TBI, training in problem-solving, communication, and self-awareness/regulation, and exercises to reinforce understanding. After completing the online

For peer review only - http://bmjopen.bmj.com/site/about/guidelines.xhtml

modules on their own, families meet with a trained therapist to implement to the problem-solving process around an Aim (goal) that they have identified. Thus, the online problem-solving program was grounded in the scientific literature, tailored to address the unique neurobehavioral and family consequences of TBI, and refined through stakeholder input[20]. Problem-solving therapy has also been trialed as a telephone-based intervention for adults with mild TBI and with families of adults with more significant brain injury[24], suggesting its potential utility across age groups and modes of delivery[25].

Research to date has been undertaken in the United States, and has focused on children and adolescents who have survived a TBI and their families[20, 26-31]. These studies have demonstrated improvements in EF, child behaviour, parental depression and family-child conflict when comparing TOPS with an internet-resource comparison control. Families have reported finding the online delivery of the intervention helpful, making the intervention easy to access at a time that is convenient for them. This is an important consideration when providing interventions for children with pABI in the UK and their families, because brain injury services in the UK often cover a large geographical region (many accepting national referrals), and families often have multiple commitments when caring for these teenagers with complex needs.

Despite this emerging evidence base, it is not yet known whether the gains described can also be demonstrated in adolescents with non-traumatic brain injuries (e.g. brain tumour, stroke), when the intervention is delivered remotely (i.e. no initial home visit to set up the intervention thus keeping delivery costs to a minimum), and if the intervention is cost-effective relative to treatment as usual (TAU).

A British version of the TOPS programme, (TOPS-UK) has been developed. The didactic material is presented with British English spelling and narration, UK-based information including scenarios, video clips and resource links. TOPS-UK has been further modified to include examples of those with paediatric acquired brain injury as well as traumatic brain injury.

The potential of TOPS-UK to have a positive effect on the lives of thousands of adolescents with pABI needs testing in a rigorous multi-centre, randomised, controlled trial (RCT) in the UK, allowing a definitive test of its clinical and cost effectiveness. This feasibility study is designed to determine if a large RCT is feasible, with the ultimate aim of the research being to provide an evidence base for effective neuropsychological interventions to be recommended by the Department of Health guidelines (e.g. NICE) for survivors of pABI.

A two-arm randomised controlled feasibility phase II study will be conducted[32]. The aim is to assess whether a larger, fully powered, definitive randomised controlled trial (RCT) and cost-effectiveness analysis can be successfully planned and delivered. In order to gain as much information as possible to inform a fully powered RCT, additional objectives are considered in this feasibility trial, including accessing information related to treatment set-up, delivery costs and resource use (Table 1).

Table 1 Study objectives

Number	Study objectives
1	Are clinicians able and willing to identify participants (e.g., has time and access to database, medical records or clinic lists to identify potentially eligible patients)?
2	Are the online screening, consent, and randomisation process feasible?
3	How many participants at each site are initially eligible, approached and consented, have completed screening, and baseline questionnaires, been randomised (with a recruitment target of at least 5 participants per site), completed treatment completed outcome

For peer review only - http://bmjopen.bmj.com/site/about/guidelines.xhtml

	assessments?
4	Do adolescents and parents find the intervention and outcome measures acceptable?
5	What are parents' and adolescents' experiences of study participation?
6	Is it possible to calculate means and standard deviations for a full RCT for the potential primary outcome measure?
7	What resources will be required to run a main trial?
8	Do some sites need additional support to be able to recruit to target in a future trial?
9	Do the trial and economic evaluation methods and procedures yield the information required?
10	How willing are participants to be randomised and complete outcome measures?
11	How well do participants adhere to the intervention (number of sessions completed)?
12	How many complete and analysable datasets are yielded, and what is the level of missing data?
13	How many participants are lost to follow up?
14	What is the coach's experience of supporting intervention delivery?
15	What are adolescent and parent experiences of TAU? (TAU arm)

METHODS AND ANALYSIS

This is a randomised, controlled, multi-centre feasibility study in young patients with pABI aged 12-18 years. Twenty-five participants will be randomised (minimised by site and type of brain injury) in a 1:1 ratio to receive TAU or treatment as usual plus Teen Online Problem-Solving (TAU+TOPS-UK). Treatment allocation will not be blinded. The protocol (version 4.1, 30 August 2018) is registered on Current Controlled Trials ISRCTN10906069 and follows Standard Protocol Items, Recommendations for Interventional Trial (SPIRIT) guidelines[33].

Setting

Two settings will be utilised to examine feasibility outcomes associated with recruitment of adolescents known to have had a pABI:

1. Direct referral by clinicians of adolescents who are under the outpatient care (usually neurology, neuropsychology or paediatric) of five participating NHS Trusts in England. Screening logs will be maintained at all sites to record the number of patients screened and enrolled in the study. These sites were selected to be representative of the range of types of services which might be included in a future trial, with TAU ranging from no provision through to services providing multidisciplinary out-patient care over the long-term.

2. Recruitment from Participant Identification Centres (PIC) in primary or secondary care, or charitable organisations. Potential participants will respond directly to study promotion including leaflets/posters/participant information sheets distributed by parent-led groups, charities or support groups.

Study participation will be supported by research nurses at each participating NHS Trust. For participants directly referred to the study, nurses will make the first approach to the family. For those identified through PICs, families will be asked to contact the research nurse whose details are provided on the patient information sheet (PIS). The research nurse will support all families during the online consent and will be a point of contact for all participants during the study. The research nurse or other research team member will support baseline and follow-up measure completion. The intervention will be provided through online materials and video conference links with a coach trained in the TOPS-UK intervention. The coach will also receive weekly supervision from a qualified

For peer review only - http://bmjopen.bmj.com/site/about/guidelines.xhtml

clinical psychologist on the research team to ensure treatment fidelity. The single study coach will be centrally based at the lead site.

Participant inclusion criteria

To be included in the study, participants will be aged 12-18 years at the time of recruitment and have survived a pABI. Diagnosis of pABI includes: moderate to severe TBI (Glasgow Coma Scale score <12 and/or post-traumatic amnesia > 24 hours and/or loss of consciousness >30 minutes), stroke, brain tumour, central nervous system infection (fungal, bacterial protozoal or viral origin including encephalitis, meningitis, brain abscess, tuberous sclerosis, acute disseminated encephalomyelitis and Guillain-Barre syndrome). Participants must be medically stable (having reached a plateau in recovery following the index injury or illness). They must also have access to the internet, and have executive function (EF) difficulties in the opinion of the local Principal Investigator (PI). The local PI is not required to undertake any EF assessment for the purposes of the study, but will use their clinical judgement, and infer from any assessments that they have undertaken, whether a participant is likely to be experiencing EF difficulties in their everyday lives. At least one parent or guardian living with the adolescent must be available for the family to participate. Adolescents with a co-morbid diagnosis of attention deficit hyperactivity disorder, autistic spectrum disorder or specific learning difficulties (e.g. reading difficulties including dyslexia) will be eligible to be recruited.

Participant exclusion criteria

Participants will be excluded from the study for the following reasons: insufficient English language, capacity or willingness for the parent/adolescent to consent/assent to the study; pre-injury or co-morbid conditions such as sensory impairments and global developmental delay, known to impair engagement with the computer and treatment materials; or non-accidental brain injury.

Participant recruitment

The trial complies with the Declaration of Helsinki and Good Clinical Practice (GCP) guidelines. GCP compliance ensures that the rights, safety and wellbeing of research participants are protected and that research data are reliable. At sites, the local PI, research nurse or other member of the research team in conjunction with the clinical team will identify potential eligible participants, usually from current outpatient clinic lists or review of medical records. An anonymised log of all participants screened with reasons for exclusion will be kept at site. Information sheets for both adolescents and their parents/guardians will be provided to potentially eligible families. The relevant PIS will be given in person to families attending an outpatient clinic, or sent by post with a cover letter from the recruiting clinician. All families will be provided with contact details and informed that a member of the research team will contact them by telephone at least 24 hours after receipt of the information to discuss the study.

During this telephone call, the parent and adolescent will be given the opportunity to ask questions about the study and to confirm whether they are interested in participating. If interested, eligibility criteria will be checked with the parent to confirm suitability for the study and ascertain which parent will participate in the intervention and complete parent-rated outcomes. A summary of what to expect from the on-line screening and consent process will also be provided.

The process of explaining the study and determining eligibility of potential participants will be undertaken by an appropriately trained member of the research team as delegated by the PI, depending on local arrangements. All staff undertaking this process for this study must have completed GCP training provided by a responsible organisation (e.g., University, NHS Trust) and must be authorised by the PI to explain the study and assess eligibility, on the site's study delegation log.

For peer review only - http://bmjopen.bmj.com/site/about/guidelines.xhtml

Online Screening and Consent

Informed consent and assent will be obtained via the study-specific website, developed and maintained by the UK Clinical Research Collaboration (UKCRC)-registered Peninsula Clinical Trials Unit (CTU) at Plymouth University.

Consent/assent

The consent process will depend upon the age of the participants:

- Participants aged 16-18 years will provide online informed consent
- Participants aged 12-15 years will provide online, informed assent. Those who have given assent and who reach the age of 16 years during the study, will be asked to provide online informed consent
- Parents of all participants aged 12-15 years will provide online informed consent on behalf of their child ('signature' from one parent required)
- All participating parents will provide online informed consent for their own participation in the study

Web-based consent process

The research nurse will enter on the study website brief details of those families who wish to participate in the study and who satisfy the initial inclusion/exclusion criteria. This will trigger an email to the adolescent, containing a link to the age-appropriate assent or consent form within the website, which the adolescent should complete.

Following completion of the adolescent assent/consent form, a separate email will be sent to the nominated parent participant containing a link to the parental consent form. Parents of adolescents aged 12-15 years will be asked to consent on behalf of their adolescent in addition to giving consent for their own participation. Two reminder emails will be sent from CTU followed by a 'last chance' email, all with reminders of the study link. If the family has not completed the consent process after two weeks, the local research team will be notified and will contact the family to ask if they still wish to participate in the study.

Face-to-face consent option

If the parent and/or adolescent has not completed the online consent process, CTU will inform the local research nurse who will telephone the family to offer the option of a clinic visit when the nurse can support the parent and/or adolescent to complete online consent/assent.

Online BRIEF-2 parent-rating and allocation of study number

Following completion of the consent process, the parent will be prompted to complete the online Behaviour Rating Inventory of Executive Function (BRIEF-2[34]) parent-rating questionnaire. If the parent does not wish to complete this immediately following the consent process, it will be possible to return to the website to complete it later. Two reminder emails will be sent from CTU followed by a 'last chance' email as above. Face-to face support to complete the BRIEF-2 will also be offered by the research nurse, as above. The parent must complete all elements of the BRIEF-2 before progressing to the baseline measures as this is the proposed primary outcome measure for a definitive trial.

The website will calculate the BRIEF-2 scores. The website will assign a unique 4-digit study number to each participant and participants will be identified in all study-related documentation by this study number. An email to both the adolescent and the parent will then be sent, inviting them to follow a web link to complete the baseline measures, and informing them of their study number.

For peer review only - http://bmjopen.bmj.com/site/about/guidelines.xhtml

As part of the consent process, adolescents and parents will be reminded via the website that they are free to withdraw from the study at any time without giving a reason and without affecting further treatment.

Baseline data collection (adolescent and parent self-completion)
On receiving the link to the baseline measures page of the study website, the adolescent and parent will complete their separate baseline questionnaires. The baseline measures should be completed within two weeks; if either the parent or adolescent have not undertaken these after one week, an email reminder to the parent (in all cases) and the adolescent (if adolescent measures not done) will be sent. In addition to the questionnaires, the parent will provide sociodemographic information, details of the adolescent's past medical history and concurrent medication.

Participants will also be given the option of completing baseline measures with telephone support from a member of the research team. A £15 gift voucher will be sent to each family upon completion of their baseline measures.

Randomisation
Participants will be randomised via a web-based system created by the CTU in conjunction with the trial statistician, using minimisation by study site and type of brain injury (TBI/tumour/other). Participants will be randomly allocated in a 1:1 ratio to receive either treatment as usual (TAU) or TOPS-UK + TAU. The minimisation process will retain a stochastic element to retain allocation concealment. If any participant is found to be ineligible following randomisation, he/she will be excluded from analyses.

This study is not blinded. Following randomisation, the adolescent, parent, PI and research nurse will be notified by email of the adolescent's treatment allocation, and this information will be held on the study site file. The parent and adolescent will also be advised when to expect further contact from the research team. In addition, the TOPS coach will be notified of all participants allocated to the intervention arm (TOPS-UK + TAU).

Baseline data collection (research nurse)
The research nurse or another member of the research team at each site will record relevant injury characteristics data for each adolescent (including premorbid/developmental history) in the study-specific web-based case report form (CRF).

Recording study participation in medical notes
The research nurse will make a record of study participation in the adolescent's hospital notes according to local practice, stating that consent was obtained online and face-to-face support was given if appropriate. The research nurse will file a copy of the PIS in the hospital notes along with printed evidence from the study website of the informed consent process and who was involved. The nurse will also send a standard letter to the participant's GP recording study participation.

INTERVENTION
The study has two treatment arms, 'TAU' and 'TAU+TOPS-UK'. Those allocated to the TAU+TOPS-UK arm will work through the intervention programme taking 1-2 weeks per module. One coach will be trained to deliver the intervention to all of the participants in the intervention arm. The coach will coordinate the timing of the modules so that all families will have completed the intervention at 16 weeks. This will allow time for other commitments and holidays/breaks to be built into the schedule. The length of time taken to complete each module will be recorded to inform timing of follow-up in the main trial.

For peer review only - http://bmjopen.bmj.com/site/about/guidelines.xhtml

Treatment as usual (control)

TAU will vary at each recruitment site because currently there is no evidence-based treatment for adolescents with pABI and their families. The type of TAU received will be recorded for each participant at baseline via a parent-rated measure of adolescent health and social care resource use (a modified version of the Client Service Receipt Inventory). There will be no opportunity for any TAU participants to receive the TOPS-UK intervention at any point during or after the study.

Teen Online Problem-Solving – TOPS (intervention*)*

TOPS is an online intervention, which is provided by the Cincinnati Children's Hospital website, via a link from the study website. For the purposes of this study, the US-based TOPS treatment content was edited to include British (English) spelling, narration and UK resource links, and modified to suit participants with pABI rather than traumatic brain injury only. All participants allocated to the TAU+TOPS-UK arm will be supported by a single TOPS-UK coach who will make weekly contact by video conference.

For those participants allocated to TAU+TOPS-UK, the TOPS-UK coach will contact the parent and/or adolescent by telephone, introduce themselves, discuss how to access the treatment modules on the website and how to log in for subsequent video call sessions. The coach will email these participants a 'start-up' pack describing how to access the online materials.

Ten subsequent sessions (Table 2) consisting of self-guided didactic content regarding problem-solving skills, video clips modelling these skills, and exercises to practise the skills, will then be completed every 7-10 days by the family and the adolescent with pABI. The online modules should take 30 minutes to complete, with the video call sessions taking 60 minutes. The parent will work together with the adolescent to complete the sessions. When the family has reviewed the materials for each session, the TOPS-UK coach will conduct a video call with the adolescent and parent who agreed to participate in the study. During this session they will review the online materials and practise the problem-solving skills using a problem identified by the family/adolescent. They will then plan the next session and agree a suitable time for the video call. Ideally, video calls will be held weekly, but this period can be increased as agreed through discussions between the coach and the family.

For all families allocated to TAU+TOPS-UK, the TOPS-UK coach will record details in the study database relating to intervention compliance e.g. number and date of Skype sessions completed, length of each session, progress/engagement of adolescent and parent.

Table 2 TOPS-UK sessions (complete 10 in total)

**Core sessions (complete all five)**	Getting started and staying positive
	Steps of problem-solving
	Getting organised
	Staying in control
	Taking care of you
**Tailored sessions (choose four)**	Dealing with fatigue
	Managing fear and worry
	Controlling your anger and improving communication
	Listening, talking and reading non-verbal cues
	Social behaviour and joining a group
	Working with the school
**Core final session**	Bringing it all together

For peer review only - http://bmjopen.bmj.com/site/about/guidelines.xhtml

Completion of all ten modules is expected to take each family 16 weeks in total. If the programme has not been completed after 16 weeks the intervention will be discontinued at that point. Study follow-up will proceed as if the programme had been completed.

Sample Size Calculation

The study will aim to screen 20 potential participants at each site (n=100 in total), and aim to recruit a sample size of 25 participants from five sites. This should provide sufficient data to assess the feasibility and acceptability of the study. Although the sample size for the full RCT will be estimated based on the minimum clinically important difference (MCID) for the BRIEF score[35] (5-points), the standard deviation for this patient population is currently unknown. A sample size of 25 is considered a realistic target and would be sufficient to address the feasibility aims (i.e. acceptability of questionnaires etc), as well as aiming to provide at least 12 participants reporting quantitative baseline data to calculate the required standard deviations[36].

Data Management

The CTU data management team is responsible for data management. Each participant will be allocated a unique trial number on consenting to participate and will be identified in all study-related documentation by the trial number and initials. A record of names and addresses linked to participants' trial numbers will be maintained by the research nurses at each site for administrative purposes and stored securely. This is an online study, with no requirement for data entry at CTU. Functions within the study website will remind participants to complete online measures, and flag up missing fields. However, participants will be able to progress through a questionnaire leaving data fields unfilled. The SQL Server database will be designed and maintained by the CTU data programming teams. Access by researchers will be password protected. In order to avoid problems with mislaid usernames and passwords, participants will access the web pages through links emailed to them by CTU. Once a web page has been completed by the participant it will be locked to prevent further data entry.

Confidentiality

All data will be collected and managed in accordance with the Data Protection Act 1998. Each participant will be allocated a unique study number and will be identified in all study-related documentation by their study number and initials. All data will be entered on a password-protected SQL Server database and encrypted using a stored procedure. After all data cleaning has been performed and the database locked, anonymised data will be exported to the trial statistician.

Data Analysis Plan

All randomised participants will be included in the analyses according to their randomised allocation, irrespective of adherence to treatment in the TOPS-UK arm or receipt of treatment in the TAU arm. No imputation of missing baseline or follow-up data will be performed. The study is not sufficiently powered to detect a significant treatment effect with regard to clinical or cost-effectiveness and thus a formal comparison will not be undertaken. The reported analyses will therefore be restricted to descriptive statistics on the outcome measures with appropriate point estimates (mean, median, percentage, etc.), standard deviations, and 95% confidence intervals for between group differences. For the questionnaire outcomes, approaches to missing individual items will be in accordance with the guidelines for missing item procedures for each questionnaire. Where no guidelines for individual missing items are available, the mean of the completed items will be used to replace missing items if 10% or fewer are missing. Statistical analyses will be performed following final data cleaning and locking of the dataset. No interim analyses are planned. All analyses will be performed using Stata v.14 and will be performed by a statistician using a dataset with treatment allocation masked.

For peer review only - http://bmjopen.bmj.com/site/about/guidelines.xhtml

Assessment of trial feasibility

The primary feasibility outcomes of this study include: (i) number of participants at each stage of the study, at each site, with stages including: identified as potentially eligible, approached, consented to study, completed screening and baseline, randomised (with at least five participants being recruited at each site), completed treatment, and completed outcome assessments; (ii) any difficulties experienced at sites that may affect their ability to recruit in future will be identified and reviewed; (iii) evaluation of appropriateness of the trial and economic evaluation methods and procedures; (iv) assessment of participants' willingness to be randomised to treatment allocation; (v) adherence to treatment assessed as number of sessions completed, a session will be recorded as complete if the participant has been through every page, reached the end and completed the coach web-linked session related to that material (with a target of participants completing at least five sessions); and (vi) attrition (with a target of at least 80% of participants completing follow-up assessments).

We will report the proportion of screened families who are found to be eligible, the proportion of eligible families who are recruited and randomised to their allocation, and the proportion of randomised participants who provide outcome data at follow-up, with 95% confidence intervals. Our sample size of 25 participants will allow us to estimate loss to follow-up (anticipated to be 20%) with a 95% CI of +/- 13 percentage points. The further outcome is evaluation of the standard deviation of the BRIEF-2 (parent) score for this patient population. This is the proposed primary outcome for the main trial and required for calculation of the sample size[36].

Measurement of outcomes

This feasibility study aims to evaluate all aspects of the proposed fully powered RCT and cost-effectiveness analysis including recruitment and retention numbers, and completion of outcome measures proposed for the main trial. Outcomes will be assessed by questionnaires and interviews. Table 3 provides a summary of outcome measures for this feasibility trial.

Table 3. Summary of Outcome Measures

For peer review only

For peer review only - http://bmjopen.bmj.com/site/about/guidelines.xhtml

Outcome group	Outcome measure	Objective	Evaluation time point(s)
Primary outcome (main trial)	Parent BRIEF-2	6	Baseline, 17 weeks post-randomisation
Secondary outcomes (main trial)	Adolescent: RCADS, SDQ, CBQ, BRIEF-2, CHU9D<br>Adult: RCADS, SDQ, EQ5D-5L*, PHQ9*, GAD7*, CBQ, CSRI	1,10,11	Baseline, 17 weeks post-randomisation
Measures of adherence (intervention group)	Participation in weekly Skype sessions.<br>Record of login to intervention website (frequency, duration, progression)	3,11	Data recorded by coach.<br>Data captured automatically by database throughout 16 week intervention period.
Intervention feasibility and acceptability (intervention group)	Qualitative interviews with families<br>Intervention acceptability questionnaires (adolescent and parent)<br>Qualitative interview with coach	1-4, 6, 9	End of trial<br>17 weeks post-randomisation<br>End of trial
Experience of TAU (TAU arm)	Qualitative interviews with families	15	End of trial
Study acceptability (both groups)	Study participation questionnaires (adolescent and parent)<br>Qualitative interviews (adolescent and parent)	5	End of trial

Abbreviations:

BRIEF-2 Behaviour Rating Inventory of Executive Function, 2nd Edition
RCADS Revised Child and Anxiety Depression Scale
SDQ Strengths and Difficulties Questionnaire
CBQ Conflict Behaviour Questionnaire
CHU9D Child Health Utility 9D
EQ5D-5L* EuroQol 5 Dimensions 5 Levels Questionnaire
PHQ9* Patient Health Questionnaire 9
GAD7* Generalised Anxiety Disorder 7-item
CSRI Client Service Receipt Inventory
*Measures relating to parental outcome

Outcome measures

These are detailed in Table 4. The proposed primary outcome for the main trial is the BRIEF-2[34], a parent and self-report measure of everyday executive function skills. Secondary outcomes will include parent reports of their child's health and behavior, using the Revised Child Anxiety and Depression Scale[37] (RCADS) and Strengths and Difficulties Questionnaire[38] (SDQ) to evaluate the adolescents' social, emotional and behavioural functioning.

A mixed methods approach (questionnaires and interviews) will be used to address outcomes relating to adolescent and parent acceptability of the intervention and outcome measures, coach's experience of supporting intervention delivery, parents' and adolescents' experiences of study participation, assessment of participants' willingness to be randomised to treatment and complete outcome measures, evaluation of appropriateness of the trial and economic evaluation methods and procedures, and adolescent and parent experiences of TAU (TAU arm).

For peer review only - http://bmjopen.bmj.com/site/about/guidelines.xhtml

Parental reports of their own and family quality of life, health, emotional functioning and family interactions will be evaluated using the EuroQol 5 dimensions questionnaire[39] (EQ-5D-5L), the Patient Health Questionnaire[40] (PHQ-9), the Generalised Anxiety Disorder[41] (GAD-7) questionnaire and the Conflict Behaviour Questionnaire[42] (CBQ).

Use of health and social care resources will be assessed through parental report on the Modified Client Service Receipt Inventory[43] (CSRI). Adolescents involved in the study will also complete questionnaires relating to their executive function skills (BRIEF-2[34] adolescent version), quality of life (Child health-related quality of life[44]; CHU-9D), emotional functioning (RCADS[37]), social, emotional and behaviour functioning (SDQ[38]) and their family interactions (CBQ[42]).

The EQ-5D-5L and CHU-9D also provide data on quality-adjusted life-years (QALYs) which can be used in cost-effectiveness analyses.

Table 4 Trial Schedule

Study procedure	Screening	Baseline	16 week intervention period	Follow-up 17 weeks
Consent/assent (adolescent +/- parents)	X			
Consent (parents)	X			
Characteristics of ABI (CRF)	X			
Current medication (adolescent)		X		X
BRIEF parent-rating	X			X
**Parents**				
Demographics, past medical history		X		
SDQ parent-rated		X		X
PHQ-9		X		X
GAD-7		X		X
CBQ		X		X
EQ5D-5L		X		X
CSRI		X		X
RCADS parent version		X		X
**Adolescents**				
Consent/assent (participants +/- parents)	X			
BRIEF-2		X		X
RCADS		X		X
SDQ		X		X
CHU-9D		X		X
CBQ		X		X
**Both parent and adolescent**				
Treatment adherence				X
Treatment acceptability rating				X

For peer review only - http://bmjopen.bmj.com/site/about/guidelines.xhtml

Study participation feedback				X
Qualitative telephone interviews (all)				X

Follow-up questionnaires

Adolescents and their parents in both treatment arms will be sent an email asking them to complete their online follow-up outcome measures at 17 weeks post-randomisation. In the event of non-completion, at least two reminders will be sent by email, followed by a reminder telephone call from the site research team if required. As with the baseline measures, participants will be given the option (at consent/assent) to complete the online follow-up measures with telephone support from a member of the research team. If participants do not complete the follow-up measures within four weeks they will receive a 'last chance' email with a reminder of the study link. A £15 gift voucher will be sent to each family following completion of the follow-up questionnaires.

It is acknowledged that a definitive trial would require longer follow-up than this feasibility study will provide, but the feasibility trial will inform the acceptability of outcome measures and the experience of participating in the trial. The power calculation for a definitive trial with longer follow-up will account for a higher level of attrition than this feasibility trial.

QUALITATIVE INTERVIEWS

A single qualitative researcher will conduct semi-structured qualitative process evaluation interviews by telephone or video call with all participants (including those who have not completed follow-up questionnaires but who have not formally withdrawn from the study), research nurses and the study coach. PIS relating to the qualitative interviews will be emailed in advance and consent will be sought at the beginning of the interview by the qualitative researcher. Adolescent and parent participants will be offered the option of being interviewed together or separately. Interviews will be conducted with the help of an agreed topic guide (see online supplementary appendix) and are expected to last between 20-60 minutes. Interviews will be audio-recorded and transcribed verbatim. The transcribed interview data will be fully anonymised and any demographic data about participants will be stored separately. Qualitative interview data will be managed using a computer software package such as Nvivo 11 and thematically analysed[45]. The analysis and results will be checked/validated by a second qualitative researcher. The following interviews will be conducted:

1. **All participants** (both groups). These interviews will allow participants to describe their whole experience of participating in the study including their willingness to be randomised and their experiences and acceptability of outcome measures, what worked well and what less well. This will cover aspects such as clarity of PIS, acceptability and ease of completing the online consent process, ease of completing online questionnaires and the parental support required the adolescent to participate and remain engaged. The interviews will explore adolescent and parent experiences of TAU or TAU+TOPS-UK, including parent's perceptions of the impact of TAU/TAU+TOPS-UK on their ability to support the adolescent, adolescent's and parent's perceptions of the impact of TAU/TAU+TOPS-UK on adolescent's ability to stay positive, solve problems, be organised, control their emotions and look after themselves (self-care), and participants' views on potential improvements to TAU/TAU+TOPS-UK. In addition, participants in the TAU+TOPS-UK will be asked about their experience of working with the coach via video calls. This information will be helpful when planning the full RCT and implementation studies. After completion of the interview, each family will be sent a £15 gift voucher as a token of appreciation.

2. **Interview with TOPS-UK coach.** A single interview will explore the coach's experiences of supporting the TOPS-UK intervention. The interview will be held by telephone, or face to

For peer review only - http://bmjopen.bmj.com/site/about/guidelines.xhtml

face, once every participant allocated to the intervention arm has completed the programme.

3. **Interviews with research nurses.** All research nurses involved with the study at the five participating sites will be invited to participate in a single interview to explore their experiences of supporting families through the consent process and any support required to complete baseline or follow-up measures. Feedback on methods of contact with families, number of contact attempts made, how much support was required from adolescents and/or parents, barriers to recruitment and suggestions for future studies will also be sought.

Participants who withdraw from the study, participants who register their interest but do not complete the study consent forms, and participants who discontinue the intervention and who do not complete the follow-up questionnaires (i.e., non-adherent but not explicitly withdrawn), will be invited to complete an anonymous online feedback survey. The survey will invite participants to comment on any aspects of the study that they found difficult, any aspects of the study that they liked, and suggestions on how the study can be improved.

ECONOMIC EVAUATION

This feasibility study will be used to develop a framework for a subsequent, policy-relevant, cost-effectiveness analysis to be undertaken alongside a future RCT. Economic evaluation methods will be developed and assessed regarding the collection of resource use, cost, and outcome data. Data on resource use associated with the set-up and delivery of the TOPS treatment will be collected at the participant and coach level e.g. coach contact and non-contact time per participant, equipment and consumable costs, training and supervision requirements for the TOPS-UK coach. Data regarding service use will be collected from all participants' parents using a version of the CSRI specifically modified for this population. This includes use of mental health, community rehabilitation, and neuropsychology and educational psychology services and has been previously trailed in this population (Wilson et al., in preparation). In addition to informing the development of the economic evaluation methods, this will provide a profile of the resources and services that constitute TAU. The study will also consider the most appropriate manner in which to capture data on health-related quality of life for use in the estimation of the cost per QALY (quality-adjusted life year) of the TOPS-UK treatment. The feasibility of participants self-completing the CHU-9D and parents completing the EQ-5D-5L, will be assessed.

In addition to this primary economic outcome, the appropriateness of considering the cost-per unit change on other relevant indicators of health status collected in the baseline and follow-up phases will be explored.

SAFETY REPORTING

The risks associated with participating in this study are considered minimal. There is a slight chance for those in the intervention group that raising awareness of injury-related cognitive or behavioural problems through communication and problem-solving might increase family burden and possibly contribute to conflict between family members. However, the purpose of the intervention is ultimately to equip families with skills to handle these difficulties by learning how to change the way they solve problems and talk with one another, and the TOPS-UK coach supporting the online intervention will be trained to handle any emerging problems. Should any issues arise, the coach will have access to a qualified clinical psychologist to provide further support and advice. There is therefore no requirement to report non-serious adverse events in this study, although serious adverse events (SAEs) will be monitored.

For peer review only - http://bmjopen.bmj.com/site/about/guidelines.xhtml

SAEs may be reported by clinicians or researchers at site, the TOPS-UK coach, by participants themselves or by any other informant. Adverse event data will be monitored by the Trial Steering Committee (TSC) to ensure safety. The TSC includes an independent statistician, psychologist, paediatrician and patient and public representative and will meet on approximately four occasions. All suspected SAEs will be reported within 24 hours of discovery to the CTU who will notify the Chief Investigator. All SAEs will be followed up until resolution.

PARTICIPANT AND PUBLIC INVOLVEMENT

A PPI group including patients and the CI was convened at an early stage of grant development and trial design and continued to meet during study set up. The group gave feedback on the patient-facing materials including age-appropriate PIS, informed consent forms and qualitative interview topic guide content. A lay-representative who is Director of Services and Innovation at the national Child Brain Injury Trust (CBIT) is a co-applicant to the grant, and a member of both the Trial Management Group (TMG) and TSC. During the recruitment period, this lay representative has been liaising with local CBIT representatives and PICs to identify potentially eligible families. A study twitter account will post trial updates to promote public engagement with the study. The twitter site currently follows and is followed by national brain injury charities disseminating trial updates to a wide audience.

Another PPI representative (parent of a child with pABI) is a member of the TMG, regularly attending meetings, and freely contributing to the discussions. Both members provide advice and suggest solutions to problems encountered during the trial, with particular expertise in barriers to recruitment and communication issues within families with a teenager with pABI. This expertise has been cascaded to site staff through a research nurse forum.

The contribution of PPI members within the TMG and TSC will be valuable during analysis, interpretation and dissemination of the study results. Specifically, PPI members will be asked to contribute to data interpretation, assess whether the feasibility study has met its objectives, and support different approaches to dissemination of the study. They will also be asked to comment on potential changes for the main trial. The Director of Services and Innovation at CBIT will also support wider PPI in the development of the main trial.

STUDY MANAGEMENT AND OVERSIGHT

The study sponsor organisation is the Royal Devon and Exeter NHS Foundation Trust, Barrack Road, Exeter EX2 5DW. Day to day trial management is administered through the UKCRC-registered Peninsula Clinical Trials Unit at Plymouth University. A Trial Management Group including the Chief Investigator, CTU trial managers, trial statistician and other personnel relevant to the study (e.g. clinicians, CTU data manager, patient and Sponsor representatives) will meet regularly (usually monthly) throughout the duration of the trial to oversee practical management of the trial.

A TSC, chaired by an independent member, will oversee the conduct and safety of the trial, ensuring that milestones are achieved and general scientific probity is maintained. A Data Monitoring Committee was not required for this feasibility study.

ETHICS AND DISSEMINATION

The study will be undertaken at acute NHS Trusts, subject to appropriate Research Ethics Committee (REC) and Health Research Authority (HRA) approvals. The trial will be conducted in accordance with the protocol, the principles of the Declaration of Helsinki and ICH GCP. Any amendments of the protocol will be submitted to the REC for approval. On request the Chief/Principal Investigator

For peer review only - http://bmjopen.bmj.com/site/about/guidelines.xhtml

should make available relevant trial-related documents for monitoring and audit by the Sponsor or the relevant Research Ethics Committee.

The study team will prepare a plain English summary of the study results which will be sent to the study participants as soon as possible after the end of the study. The final results of the study will be disseminated via presentations at appropriate scientific meetings and conferences and publication in appropriate peer-reviewed journals. The data from this study will be used to inform the design and accompanying grant application for a fully powered RCT should the study be considered feasible.

DISCUSSION

In our opinion, TOPS-UK has genuine potential to have a profound and positive effect on the lives of thousands of adolescents with brain injury. The intervention itself already exists and has demonstrated efficacy in the United States, including gains in executive function (planning, problem-solving), reductions in behaviour and mood difficulties, and reductions in family burden and stress. TOPS-UK urgently requires testing in a rigorous multi-centre randomised trial in the UK, allowing a definitive test of its clinical and cost effectiveness.

The importance of the current study is to determine if a large RCT is feasible (e.g., recruitment, access to resource use data, appropriate outcome measures etc). In addition, although the study is not powered to detect effectiveness, individual patients participating in the study might gain direct benefit from the treatment (TAU+TOPS) in terms of increased executive function skills, reduced behaviour and mood difficulties, and improved quality of life. The feasibility study might also have some implications on clinical and public health practice by raising awareness of pABI, the need for effective treatment, and the variability of TAU (via the dissemination workshop and reports), and improve the measurements of quality of life and health status in adolescents with pABI (via conferences). If the current study demonstrates feasibility, outcomes will be used to inform the development of a fully powered phase III RCT to examine effectiveness and cost-effectiveness of TOPS-UK.

If the phase III RCT is successful, then the clinical and public health practice developments are potentially far reaching. For example, in addition to the direct benefits to the adolescent and his or her family, TOPS-UK might also prevent considerable NHS costs in the future by reducing healthcare service use. Furthermore, given the increased risks of criminal behaviour and poor educational and vocational outcomes following pABI, often associated with poor executive functioning, TOPS-UK might have wider social benefits for these individuals, and society as a whole. In terms of potential impact on local policy-making and improvement in service delivery, TOPS-UK is potentially very cheap to deliver because it is web-based and involves regular but brief professional support that is delivered remotely (via video calls). Furthermore, given its web-based delivery that can be accessed at a time that is convenient for the adolescent and their family, TOPS-UK is less disruptive than clinic appointments, which are often delivered during school-hours. This is an important consideration for an individual with pABI because, typically, the adolescents have already missed a significant amount of school and struggle academically. By enabling free use of the materials to the NHS, clinical services might routinely provide TOPS-UK to adolescents with pABI and their families, meeting the needs of multiple families simultaneously. TOPS-UK might also provide a first-line of treatment for families experiencing distress and, therefore form part of a 'stepped-care' model of service delivery such that, families who continue to experience significant difficulties, or who are at high risk, can be referred to specialist services in a timely manner. The ultimate aim of the research programme is to provide an evidence-base for effective neuropsychological interventions to be recommended by the Department of Health guidelines (e.g., NICE) for survivors of pABI.

For peer review only - http://bmjopen.bmj.com/site/about/guidelines.xhtml

References

1. ONS. Office for National Statistics. Children, Education and Skills: Measuring National Well-being http://wwwonsgovuk/ons/taxonomy/indexhtml?nscl=Children%2C+Education+and+ Skills. 2012.
2. Hawley CA, Ward AB, Long J, Owen DW and Magnay AR. Prevalence of traumatic brain injury amongst children admitted to hospital in one health district: a population-based study. Injury. 2003;34:256-60.
3. Hayes L, Shaw S, Pearce MS, et al., Requirements for and current provision of rehabilitation services for children after severe acquired brain injury in the UK: a population-based study. Archives of Disease in Childhood. 2017;102:813-820.
4. WHO. World Report on Child Injury Prevention. 2008.
5. Fleminger S and Ponsford J. Long term outcome after traumatic brain injury. BMJ. 2005;331:1419-20.
6. All-Party Parliamentary Group on Acquired Brain Injury. Acquired brain injury and neurorehabilitation: time for change. www.ukabif.org.uk/campaigns/appg-report. 2018
7. Tomlin P, Clarke M, Robinson G and Roach J. Rehabilitation in severe head injury in children: outcome and provision of care. Dev Med Child Neurol. 2002;44:828-37.
8. McKinlay A, Corrigan J, Horwood L and Fergusson D. Substance Abuse and Criminal Activities Following Traumatic Brain Injury in Childhood, Adolescence and Early Adulthood. J Head Trauma Rehabil. 2013.
9. Max JE. Neuropsychiatry of pediatric traumatic brain injury. Psychiatr Clin North Am. 2014;37:125-40.
10. Williams WH, Mewse AJ, Tonks J, Mills S, Burgess CN and Cordan G. Traumatic brain injury in a prison population: prevalence and risk for re-offending. Brain Inj. 2010;24:1184-8.
11. Anderson V, Spencer-Smith M, Leventer R, Coleman L, Anderson P, Williams J, Greenham M and Jacobs R. Childhood brain insult: can age at insult help us predict outcome? Brain. 2009;132:45-56.
12. Anderson V, Spencer-Smith M, Coleman L, Anderson P, Williams J, Greenham M, Leventer RJ and Jacobs R. Children's executive functions: are they poorer after very early brain insult. Neuropsychologia. 2010;48:2041-50.
13. Best JR, Miller PH and Naglieri JA. Relations between Executive Function and Academic Achievement from Ages 5 to 17 in a Large, Representative National Sample. Learn Individ Differ. 2011;21:327-336.
14. Struchen MA, Clark AN, Sander AM, Mills MR, Evans G and Kurtz D. Relation of executive functioning and social communication measures to functional outcomes following traumatic brain injury. NeuroRehabilitation. 2008;23:185-98.
15. Jurado MB and Rosselli M. The elusive nature of executive functions: a review of our current understanding. Neuropsychol Rev. 2007;17:213-33.
16. Gracey F, Watson S, McHugh M, Swan A, Humphrey A and Adlam A-L. Age of injury, emotional problems and executive functioning in understanding disrupted social relationships following childhood ABI. J Social Care and Neurodisability. 2014;5:10.
17. Kurowski BG, Taylor HG, Yeates KO, Walz NC, Stancin T and Wade SL. Caregiver ratings of long-term executive dysfunction and attention problems after early childhood traumatic brain injury: family functioning is important. PM R. 2011;3:836-45.
18. Hughes CH and Ensor RA. How do families help or hinder the emergence of early executive function? New Dir Child Adolesc Dev. 2009;2009:35-50.
19. Ross KA, Dorris L and McMillan T. A systematic review of psychological interventions to alleviate cognitive and psychosocial problems in children with acquired brain injury. Dev Med Child Neurol. 2011;53:692-701.
20. Wade SL, Wolfe CR and Pestian JP. A web-based family problem-solving intervention for families of children with traumatic brain injury. Behav Res Methods Instrum Comput. 2004;36:261-9.

For peer review only - http://bmjopen.bmj.com/site/about/guidelines.xhtml

21. D'Zurilla TJN, A.M. Problem solving therapy. In: Dobson K, ed. Handbook of cognitive behavioral therapies. New York: Guilford; 2010:197-224
22. Wade SL, Cassedy AE, Fulks L, et al. Problem solving following traumatic brain injury in adolescence: Associations with functional outcomes. Arch of Phys Med and Rehabil. 2017;98:1614-1621.
23. Kreutzer JS, Stejskal TM, Ketchum JM, Marwitz JH, Taylor LA and Menzel JC. A preliminary investigation of the brain injury family intervention: Impact on family members. Brain Injury, 2009, (6); 535-547.
24. Rivera PA, Elliott TR, Berry JW, Grant JS. Problem-solving training for family caregivers of persons with traumatic brain injuries: A randomized controlled trial. Archives of Physical Medicine and Rehabilitation. 2008 May 1;89(5):931-41.
25. Bell KR, Fann JR, Brockway JA, Cole WR, Bush NE, Dikmen S, Hart T, Lang AJ, Grant G, Gahm G, Reger MA. Telephone problem solving for service members with mild traumatic brain injury: a randomized, clinical trial. Journal of neurotrauma. 2017 Jan 15;34(2):313-21.
26. Wade SL, Walz NC, Carey JC and Williams KM. Preliminary efficacy of a Web-based family problem-solving treatment program for adolescents with traumatic brain injury. J Head Trauma Rehabil. 2008;23:369-77.
27. Wade SL, Oberjohn K, Burkhardt A and Greenberg I. Feasibility and preliminary efficacy of a web-based parenting skills program for young children with traumatic brain injury. J Head Trauma Rehabil. 2009;24:239-47.
28. Wade SL, Walz NC, Carey J, Williams KM, Cass J, Herren L, Mark E and Yeates KO. A randomized trial of teen online problem solving for improving executive function deficits following pediatric traumatic brain injury. J Head Trauma Rehabil. 2010;25:409-15.
29. Wade SL, Walz NC, Carey J, McMullen KM, Cass J, Mark E and Yeates KO. Effect on behavior problems of teen online problem-solving for adolescent traumatic brain injury. Pediatrics. 2011;128:e947-53.
30. Wade SL, Walz NC, Carey J, McMullen KM, Cass J, Mark E and Yeates KO. A randomized trial of teen online problem solving: efficacy in improving caregiver outcomes after brain injury. Health Psychol. 2012;31:767-76.
31. Wade S, Stancin T, Kirkwood M, Brown T, McMullen K and Taylor H. Counselor-assisted problem solving (CAPS) improves behavioural outcomes in older adolescents with complicated mild to severe TBI. J Head Trauma Rehabil. 2014;29:10.
32. Craig P, Dieppe P, Macintyre S, et al. Developing and evaluating complex interventions: the new Medical Research Council guidance. BMJ2008;337:a1655
33. Chan A-W, Tetzlaff JM, Altman DG, Laupacis A, Gøtzsche PC, Krleža-Jerić K, Hróbjartsson A, Mann H, Dickersin K, Berlin J, Doré C, Parulekar W, Summerskill W, Groves T, Schulz K, Sox H, Rockhold FW, Rennie D, Moher D. SPIRIT 2013 Statement: Defining standard protocol items for clinical trials. Ann Intern Med 2013;158:200-207.
34. Gioia G, Isquith P, Guy S and Kenworthy L. Behavior Rating Inventory of Executive Function Second Edition (BRIEF 2). Psychological Assessment Resources. 2015.
35. Kurowski BG, Wade SL, Kirkwood MW, Brown TM, Stancin T and Taylor HG. Online problem-solving therapy for executive dysfunction after child traumatic brain injury. Pediatrics. 2013;132:e158-66.
36. Julious S. Sample size of 12 per group rule of thumb for pilot study. Pharmaceutical Statistics. 2005;4:5.
37. Chorpita BF, Moffitt CE and Gray J. Psychometric properties of the Revised Child Anxiety and Depression Scale in a clinical sample. Behav Res Ther. 2005;43:309-22.
38. Goodman R. The extended version of the Strengths and Difficulties Questionnaire as a guide to child psychiatric caseness and consequent burden. J Child Psychol Psychiatry. 1999;40:791-9.

For peer review only - http://bmjopen.bmj.com/site/about/guidelines.xhtml

39. Herdman M, Gudex C, Lloyd A, Janssen M, Kind P, Parkin D, Bonsel G and Badia X. Development and preliminary testing of the new five-level version of EQ-5D (EQ-5D-5L). Qual Life Res. 2011;20:1727-36.
40. Kroenke K, Spitzer RL and Williams JB. The PHQ-9: validity of a brief depression severity measure. J Gen Intern Med. 2001;16:606-13.
41. Spitzer RL, Kroenke K, Williams JB and Löwe B. A brief measure for assessing generalized anxiety disorder: the GAD-7. Arch Intern Med. 2006;166:1092-7.
42. Robin A and Foster S. Negotiating parent-adolescent conflict: A behavioural family systems approach. New York: Guildford Press; 1989.
43. Beecham J and Knapp M. Costing psychiatric interventions. London: Gaskill; 1992.
44. Stevens M, Clark A, Sander A, Mills M, Evans G and Kurtz D. Assessing the performance of a new generic measure of health related quality of life for children and refining it for use in health state valuation. Applied Health Economics and Health Policy. 2011;9:12.
45. Braun V, Clarke, V. Using thematic analysis in psychology. Qualitative Research in Psychology. 2006;3:77-101.

For peer review only

ACKNOWLEDGEMENTS

The authors are grateful for the support of the study sponsor (Royal Devon and Exeter NHS Foundation Trust) and the South West Peninsula NIHR Clinical Research Network. We acknowledge the contribution made by Dr Richard Tomlinson, Consultant Paediatrician, Royal Devon and Exeter NHS Foundation Trust who was a grant co-applicant and for his support during study set- up. We are also indebted to Theresa Pass, former Director of the Child Brain Injury Trust who was a grant co-applicant, and to Louise Wilkinson and members of the Child Brain Injury Trust for their continued support and advice during recruitment and study delivery.

AUTHORS' CONTRIBUTIONS

All authors except AJ and SH were co-applicants on the NIHR RfPB grant application and as such were involved in the design of this feasibility study. All authors contributed to successive drafts of this paper. JL was the CI for the first year of the study and led on developing the intervention, finalising the protocol and drafting this manuscript. SW designed the study intervention and contributed to study design. JV contributed to the study design and oversaw trial management. AJ is the trial manager, responsible for the day-to-day running of the trial, and contributed to drafts of this manuscript. FW is the trial statistician and provided expertise in the overall design of the trial. AH was responsible for the design and analysis of the economic evaluation component. JS was responsible for the design and analysis of the qualitative component. TF contributed to study design and provided advice and guidance on study delivery. SH contributed to trial management from a PPI perspective. AA was the lead grant applicant and took over as CI from JL for the second year of the study, providing clinical expertise, and drafting and revising this manuscript.

DATA SHARING

After the end of the study, information collected may be made available as an anonymised participant level dataset to other researchers under an appropriate data sharing agreement.

FUNDING and DISCLAIMER

This paper presents independent research funded by the National Institute for Health Research (NIHR) under its Research for Patient Benefit (RfPB) Programme (Grant Reference Number PB-PG-0614-34081). The views expressed are those of the author(s) and not necessarily those of the NHS, the NIHR or the Department of Health and Social Care. The funder has no role in study design or the collection, management, analysis or interpretation of data.

COMPETING INTERESTS

None declared.

Provenance and peer review Not commissioned; externally peer reviewed.

For peer review only

ONLINE SUPPLEMENTARY APPENDIX

Qualitative interview topic guide

General Introduction

- You have been invited to take part/are taking part in the TOPS-UK feasibility study.
- If this study is successful, we would like to conduct a bigger study, but before we do that we need your views and suggestions on the best way to run it.
- It is particularly important for us to learn about your experiences while you were taking part.
- Teenagers and parents may have different experiences. We want to know how it was for both of you, and we value all your answers.
- There are no right or wrong answers.
- Participation is voluntary so if at any point you prefer not to carry on please feel free to stop the interview. We can also have a break during the interview if you wish.
- Whatever you say in this interview will be confidential and used only for this research project. I will record this interview and have it transcribed (typed out) later to keep an accurate record of your views. If anything is recorded that could reveal your identity it will not be included in the written transcription which will be anonymised. If we use any quotes from your contributions in research conferences, publications or events, nothing that could identify you will be included.
- Is it OK with you to record this interview?

**Topics for all qualitative interviews:**	**Study objective number(s)**	***Prompts***
Their experience of online consent forms	ii	*Did you feel you had enough information to make your decision to give consent? Was the consent form straightforward?*
Their experience of being randomised to treatment	x	*How did you feel about being allocated to one group?*
Their experience of online research questionnaires (some families might not have completed all of the study questionnaires)	v	*Ease of completion, clarity of questionnaires, burden of completion, and convenience of online format (any alternative suggestions). Did reminders and periodic emails help/ hinder- any suggestions to change?*

**If completed at least 1 session of the TOPS-UK intervention: Topics for teenager and parent in the intervention (TOPS-UK + TAU)**	**Study objective number(s)**	***Prompts***
Their experience of the TOPS-UK intervention programme	iv, v, xi	*How did you feel about being in this group?*<br>*How did you find the computer sessions?*<br>*How did you find the Skype sessions?*<br>*How did you get on with the coach?*
The effects of the programme on the teenager's ability to stay positive, solve problems, be organised, control their emotions		

and look after themselves.		
The effects of the programme on the parent's ability to support their teenager.		
What could be changed to improve the TOPS-UK programme?	iv	*Were there any aspects of the programme which you would change?*<br>*What aspects did you find most/least helpful?*

**Topics for teenager and parent randomised to treatment as usual (TAU)**	**Study objective number(s)**	***Prompts***
Their experience of TAU	v, x, xiv	*How did you feel about being in the control group? Did it impact on your view of the study?*
The effects of TAU on the teenager's ability to stay positive, solve problems, be organised, control their emotions and look after themselves.		
The effects of TAU on the parent's ability to support their teenager.		
What support would be helpful to families of teenagers with acquired brain injury?		*Face-to-face, telephone, online, home visits, clinic visits, which therapists/therapy would be helpful?*

**For all participants:**<br>**Topics not already covered:**	**Study Objective number(s)**	***Prompts***
What worked well and what less well in the study?	v, x,	
Is there anything that we can do to make the study more acceptable to families like yours in the future?		
Is there anything else you would like to talk about that we haven't mentioned?		
Give contact details for further information/discussion		

Ending questions

Is there anything you would like to talk more about? Have we missed anything?

Thank participants for taking time to join the telephone interview. Explain that if anyone has any follow up questions or comments they are welcome to call/ email the researcher.

For peer review only

Reporting checklist for protocol of a clinical trial.

Based on the SPIRIT guidelines.

Instructions to authors

Complete this checklist by entering the page numbers from your manuscript where readers will find each of the items listed below.

Your article may not currently address all the items on the checklist. Please modify your text to include the missing information. If you are certain that an item does not apply, please write "n/a" and provide a short explanation.

Upload your completed checklist as an extra file when you submit to a journal.

In your methods section, say that you used the SPIRIT reporting guidelines, and cite them as:

Chan A-W, Tetzlaff JM, Altman DG, Laupacis A, Gøtzsche PC, Krleža-Jerić K, Hróbjartsson A, Mann H, Dickersin K, Berlin J, Doré C, Parulekar W, Summerskill W, Groves T, Schulz K, Sox H, Rockhold FW, Rennie D, Moher D. SPIRIT 2013 Statement: Defining standard protocol items for clinical trials. Ann Intern Med. 2013;158(3):200-207

		Reporting Item	Page Number
Title	#1	Descriptive title identifying the study design, population, interventions, and, if applicable, trial acronym	1
Trial registration	#2a	Trial identifier and registry name. If not yet registered, name of intended registry	2

Trial registration: data set	#2b	All items from the World Health Organization Trial Registration Data Set	N/A
Protocol version	#3	Date and version identifier	N/A
Funding	#4	Sources and types of financial, material, and other support	18
Roles and responsibilities: contributorship	#5a	Names, affiliations, and roles of protocol contributors	1, 18
Roles and responsibilities: sponsor contact information	#5b	Name and contact information for the trial sponsor	14
Roles and responsibilities: sponsor and funder	#5c	Role of study sponsor and funders, if any, in study design; collection, management, analysis, and interpretation of data; writing of the report; and the decision to submit the report for publication, including whether they will have ultimate authority over any of these activities	14
Roles and responsibilities: committees	#5d	Composition, roles, and responsibilities of the coordinating centre, steering committee, endpoint adjudication committee, data management team, and other individuals or groups overseeing the trial, if applicable (see Item 21a for data monitoring committee)	15
Background and rationale	#6a	Description of research question and justification for undertaking the trial, including summary of relevant	2-4

For peer review only

		studies (published and unpublished) examining benefits and harms for each intervention	
Background and rationale: choice of comparators	#6b	Explanation for choice of comparators	7
Objectives	#7	Specific objectives or hypotheses	4
Trial design	#8	Description of trial design including type of trial (eg, parallel group, crossover, factorial, single group), allocation ratio, and framework (eg, superiority, equivalence, non-inferiority, exploratory)	4
Study setting	#9	Description of study settings (eg, community clinic, academic hospital) and list of countries where data will be collected. Reference to where list of study sites can be obtained	4-5
Eligibility criteria	#10	Inclusion and exclusion criteria for participants. If applicable, eligibility criteria for study centres and individuals who will perform the interventions (eg, surgeons, psychotherapists)	5
Interventions: description	#11a	Interventions for each group with sufficient detail to allow replication, including how and when they will be administered	7-9
Interventions: modifications	#11b	Criteria for discontinuing or modifying allocated interventions for a given trial participant (eg, drug dose	9

For peer review only

		change in response to harms, participant request, or improving / worsening disease)	
Interventions: adherance	#11c	Strategies to improve adherence to intervention protocols, and any procedures for monitoring adherence (eg, drug tablet return; laboratory tests)	12
Interventions: concomitant care	#11d	Relevant concomitant care and interventions that are permitted or prohibited during the trial	N/A
Outcomes	#12	Primary, secondary, and other outcomes, including the specific measurement variable (eg, systolic blood pressure), analysis metric (eg, change from baseline, final value, time to event), method of aggregation (eg, median, proportion), and time point for each outcome. Explanation of the clinical relevance of chosen efficacy and harm outcomes is strongly recommended	10-12
Participant timeline	#13	Time schedule of enrolment, interventions (including any run-ins and washouts), assessments, and visits for participants. A schematic diagram is highly recommended (see Figure)	11-12
Sample size	#14	Estimated number of participants needed to achieve study objectives and how it was determined, including clinical and statistical assumptions supporting any sample size calculations	9
Recruitment	#15	Strategies for achieving adequate participant enrolment to reach target sample size	N/A

For peer review only

Allocation: sequence generation	#16a	Method of generating the allocation sequence (eg, computer-generated random numbers), and list of any factors for stratification. To reduce predictability of a random sequence, details of any planned restriction (eg, blocking) should be provided in a separate document that is unavailable to those who enrol participants or assign interventions	7
Allocation concealment mechanism	#16b	Mechanism of implementing the allocation sequence (eg, central telephone; sequentially numbered, opaque, sealed envelopes), describing any steps to conceal the sequence until interventions are assigned	7
Allocation: implementation	#16c	Who will generate the allocation sequence, who will enrol participants, and who will assign participants to interventions	7
Blinding (masking)	#17a	Who will be blinded after assignment to interventions (eg, trial participants, care providers, outcome assessors, data analysts), and how	N/A
Blinding (masking): emergency unblinding	#17b	If blinded, circumstances under which unblinding is permissible, and procedure for revealing a participant's allocated intervention during the trial	N/A
Data collection plan	#18a	Plans for assessment and collection of outcome, baseline, and other trial data, including any related processes to promote data quality (eg, duplicate measurements, training of assessors) and a description of study	7,9,12

For peer review only

		instruments (eg, questionnaires, laboratory tests) along with their reliability and validity, if known. Reference to where data collection forms can be found, if not in the protocol	
Data collection plan: retention	#18b	Plans to promote participant retention and complete follow-up, including list of any outcome data to be collected for participants who discontinue or deviate from intervention protocols	12
Data management	#19	Plans for data entry, coding, security, and storage, including any related processes to promote data quality (eg, double data entry; range checks for data values). Reference to where details of data management procedures can be found, if not in the protocol	9
Statistics: outcomes	#20a	Statistical methods for analysing primary and secondary outcomes. Reference to where other details of the statistical analysis plan can be found, if not in the protocol	9
Statistics: additional analyses	#20b	Methods for any additional analyses (eg, subgroup and adjusted analyses)	N/A
Statistics: analysis population and missing data	#20c	Definition of analysis population relating to protocol non-adherence (eg, as randomised analysis), and any statistical methods to handle missing data (eg, multiple imputation)	9
Data monitoring: formal committee	#21a	Composition of data monitoring committee (DMC); summary of its role and reporting structure; statement of	15

For peer review only

		whether it is independent from the sponsor and competing interests; and reference to where further details about its charter can be found, if not in the protocol. Alternatively, an explanation of why a DMC is not needed	
Data monitoring: interim analysis	#21b	Description of any interim analyses and stopping guidelines, including who will have access to these interim results and make the final decision to terminate the trial	N/A
Harms	#22	Plans for collecting, assessing, reporting, and managing solicited and spontaneously reported adverse events and other unintended effects of trial interventions or trial conduct	14
Auditing	#23	Frequency and procedures for auditing trial conduct, if any, and whether the process will be independent from investigators and the sponsor	N/A
Research ethics approval	#24	Plans for seeking research ethics committee / institutional review board (REC / IRB) approval	2,15
Protocol amendments	#25	Plans for communicating important protocol modifications (eg, changes to eligibility criteria, outcomes, analyses) to relevant parties (eg, investigators, REC / IRBs, trial participants, trial registries, journals, regulators)	14,15
Consent or assent	#26a	Who will obtain informed consent or assent from potential trial participants or authorised surrogates, and how (see Item 32)	6

For peer review only

Consent or assent: ancillary studies	#26b	Additional consent provisions for collection and use of participant data and biological specimens in ancillary studies, if applicable	N/A
Confidentiality	#27	How personal information about potential and enrolled participants will be collected, shared, and maintained in order to protect confidentiality before, during, and after the trial	9
Declaration of interests	#28	Financial and other competing interests for principal investigators for the overall trial and each study site	18
Data access	#29	Statement of who will have access to the final trial dataset, and disclosure of contractual agreements that limit such access for investigators	18
Ancillary and post trial care	#30	Provisions, if any, for ancillary and post-trial care, and for compensation to those who suffer harm from trial participation	N/A
Dissemination policy: trial results	#31a	Plans for investigators and sponsor to communicate trial results to participants, healthcare professionals, the public, and other relevant groups (eg, via publication, reporting in results databases, or other data sharing arrangements), including any publication restrictions	15,16
Dissemination policy: authorship	#31b	Authorship eligibility guidelines and any intended use of professional writers	15

For peer review only

Dissemination policy: reproducible research	#31c	Plans, if any, for granting public access to the full protocol, participant-level dataset, and statistical code	N/A
Informed consent materials	#32	Model consent form and other related documentation given to participants and authorised surrogates	N/A
Biological specimens	#33	Plans for collection, laboratory evaluation, and storage of biological specimens for genetic or molecular analysis in the current trial and for future use in ancillary studies, if applicable	N/A

The SPIRIT checklist is distributed under the terms of the Creative Commons Attribution License CC-BY-ND 3.0. This checklist can be completed online using https://www.goodreports.org/, a tool made by the EQUATOR Network in collaboration with Penelope.ai

For peer review only
